# Supplementary material for: Influencing factor of COVID-19 vaccination trust and hesitancy in Wonju city, South Korea
Source: PLoS One. 2022 Nov 14;17(11):e0277016. doi: 10.1371/journal.pone.0277016 (PMC9662712; doi:10.1371/journal.pone.0277016)
Supplement: S1 File — (PDF) [file pone.0277016.s001.pdf]

Survey start time	Survey end time
___h ___m	___h ___m

Survey District Number			surveyor		-	Response number	

Questionnaire on the Healthy City Project in Wonju City

How are you?

Wonju, a healthy city, has been promoting healthy city projects since 2004 based on the spirit of the World Health Organization (WHO). This time, we would like to evaluate the business performance so far and establish long-term goals and strategies for the healthy city project in Wonju until 2025.

Accordingly, Wonju City is conducting a study with Yonsei University to investigate the COVID-19 awareness, satisfaction, and requirements of healthy city projects for citizens of "Healthy City Wonju."

We would like to request a survey on this questionnaire to collect your opinions on the development of the Healthy City Wonju Vision 2025 during COVID-19 era, so we would appreciate it if you could respond.

The contents of your response to this survey will not be used for any purpose other than research, and I promise to keep it confidential.

May 2021

Department of Health and Sports in Wonju
Yonsei University Healthy City Research Center

Contact information:
Department of Health and Sports in Wonju ☎ 033) 737 - 2853
Yonsei University Healthy City Research Center ☎ 033) 760 - 2949

Social capital

1. Please indicate the social capital question below where applicable.

Social capital		Very unlikely	unlikely	ordinary	likely	Very likely
(1)	Do the locals trust each other in your town?	①	②	③	④	⑤
(2)	Do you trust most people who live around you?	①	②	③	④	⑤
(3)	Do you trust public schools in your area?	①	②	③	④	⑤
(4)	Do you trust government offices in this area?	①	②	③	④	⑤
(5)	Do you naturally exchange greetings with the residents of this area?	①	②	③	④	⑤
(6)	Do you think it's easy to get help when someone in our community needs it?	①	②	③	④	⑤
(7)	Do you think this area is very safe?	①	②	③	④	⑤
(8)	Is there a medical institution where you can go right away when you suddenly get sick?	①	②	③	④	⑤
(9)	Are you relieved that there is a medical institution in this area where you can go right away when you are suddenly sick?	①	②	③	④	⑤
(10)	Do you feel a sense of belonging to this area?	①	②	③	④	⑤
(11)	Are you thinking of doing something with the people around you to improve this community?	①	②	③	④	⑤
(12)	Are you satisfied with living in this area?	①	②	③	④	⑤
(13)	Do you always vote in elections?	①	②	③	④	⑤
(14)	Do you think the neighbors in this area are precious?	①	②	③	④	⑤

2. Are you participating in any of the following local gatherings or organizations?
(e.g., volunteer work, youth association, senior citizens' association, women's association, parents' association, sports club, autonomous association, neighborhood association, fire brigade, religious organization)

☐ ①Yes (question 2-1) ☐②No (question 3)

2. How long have you been active in the organization above? About _______ months

3. How many people are close to you in your town besides your family? About ________ people

4. How many people are living together now? _______ people

5. Who is your current roommate? (Duplicable)
☐ ① parents ☐② children ☐③ relative ☐④ acquaintances ☐⑤ etc( )

COVID-19

※ The following are questions related to the COVID-19 epidemic since early last year (January 2020).

6. Have you or your neighborhood (family or neighborhood) experienced COVID-19 confirmation?		
☐ ① Yes	☐ ② No	☐③ I don't know

7. Have you ever been in self-quarantine?		
☐ ① Yes	☐ ② No	☐③ I don't know

8. Do you think Wonju City's ability to respond to COVID-19 is appropriate?	
☐ ① Very appropriate<br>☐ ② Appropriate<br>☐ ③ Ordinary	☐ ④ inappropriate<br>☐ ⑤ Totally inappropriate

9. What activities do you think are needed in Wonju to prevent COVID-19?<br>(Preparation of distancing guidelines, vaccination, crisis communication with citizens, support for quarantine supplies, etc.)
Activity ① ______________________________
Activity ② ______________________________
Activity ③ ______________________________

10. To what extent do you trust the vaccine?
☐ ① Very reliable ☐② Reliable ☐③ Ordinary ☐④ Unreliable ☐⑤ Very unreliable

11. Have you ever been vaccinated against COVID-19?<br>(*Vaccines: all types including Pfizer, AstraZeneca, etc.)
☐ ① Yes (▶ question 46) ☐ ② No (▶question 11-1) ☐ ③ Don't remember (▶question 12)

11-1. Are you willing to be vaccinated against COVID-19?
☐ ① I will definitely accept it ☐ ② Probably will accept it ☐ ③ Probably won't accept it ☐ ④ I'll never accept it ☐ ⑤ I don't know yet

12. Have you ever been tested for COVID-19 at public health centers, hospitals, or screening clinics?		
① Yes	☐ ② No	☐ ③ Don't remember

General characteristic

13. What's your gender?	
☐① Male	☐② Female

14. What year was your birth?
______ year

15. How tall are you now?
Height: ______ cm

16. How much do you weigh now?
Weight: __________ kg

17. Which of the following are your occupations?	
☐① Public official<br>☐② Agricultural and forestry workers<br>☐③ Self-employed businesses and service industries<br>☐④ Professional jobs (doctors, lawyers, teachers, etc.)	☐⑤ Student, repeat student<br>☐⑥ Office worker<br>☐⑦ Housewife<br>☐⑧ Not employed<br>☐⑨ Other (______________________)

18. What is your average monthly household income, including interest, rental income, and external living assistance policies?	
□① Less than 1 million won<br>□② 1 million to 1.99 million won<br>□③ 2 million to 2.99 million won	□④ 3 million to 3.99 million won<br>□⑤ 4 million to 4.99 million won<br>□⑥ More than 5 million won

19. What is your form of residence?	
□① Mansion, apartment<br>□② A detached house<br>□③ One room	□④ Dormitory<br>□⑤ Other( )

20. How long have you lived in Wonju?
__ years ___ months

The end.

Thank you for your participation.
