# Supplementary material for: Influencing factor of COVID-19 vaccination trust and hesitancy in Wonju city, South Korea
Source: PLoS One. 2022 Nov 14;17(11):e0277016. doi: 10.1371/journal.pone.0277016 (PMC9662712; doi:10.1371/journal.pone.0277016)
Supplement: S2 File — (PDF) [file pone.0277016.s002.pdf]

조사시작시간	조사종료시간
___시 ___분	___시 ___분

조사구 번호			조사자		-	응답번호	

원주시 건강도시사업에 대한 의견조사 설문지

안녕하십니까?

건강도시 원주는 세계보건기구(WHO)의 정신에 입각하여 2004년부터 건강도시사업을 추진해 오고 있습니다. 이번에 그간의 사업성과를 평가하여 2025년까지의 원주시 건강도시사업의 장기 목표 및 전략을 수립하고자 합니다.

이에 원주시는 연세대학교와 함께 「건강도시 원주」시민을 대상으로 건강도시사업에 대한 인지도, 만족도 및 요구 사항 등을 조사하는 연구를 수행중에 있습니다.

「건강도시 원주 비전2025」 건강도시사업 개발에 귀하의 고견을 수렴하고자 본 설문지에 대한 설문조사를 의뢰드리오니 응답해 주시면 고맙겠습니다.

본 설문에 응답하신 내용은 연구목적 이외에는 사용되지 않을 것이며, 절대 비밀을 유지할 것을 약속드립니다.

2021년 5월

원 주 시 건 강 체 육 과
연 세 대 학 교 건 강 도 시 연 구 센 터

연락처: 원주시 건강체육과 ☎ 033) 737 - 2853
연세대학교 건강도시연구센터 ☎ 033) 760 - 2949

사회적 자본

1. 아래의 사회적 자본 문항에 대하여 해당하는 곳에 표기해 주십시오.

사회적 자본		매우그렇지않음	그렇지않음	보통	그러함	매우그러함
(1)	귀하의 마을에서는 지역주민들이 서로 신뢰하고 계십니까?	①	②	③	④	⑤
(2)	귀하는 주위에 살고 있는 대부분의 사람을 신뢰하고 계십니까?	①	②	③	④	⑤
(3)	귀하께서 살고 있는 지역의 공립학교에 대하여 신뢰하고 계십니까?	①	②	③	④	⑤
(4)	귀하께서는 이 지역의 관공서에 대해서 신뢰하고 계십니까?	①	②	③	④	⑤
(5)	귀하께서는 이 지역의 주민들과 자연스럽게 인사를 주고받으십니까?	①	②	③	④	⑤
(6)	우리 지역사회에서 누군가가 도움을 필요로 할 때, 쉽게 도움을 받을 수 있다고 생각하십니까?	①	②	③	④	⑤
(7)	귀하께서는 이 지역이 아주 안전하다고 생각하십니까?	①	②	③	④	⑤
(8)	갑자기 아플 때 바로 갈 수 있는 의료기관이 있습니까?	①	②	③	④	⑤
(9)	갑자기 아플 때 바로 갈 수 있는 의료기관이 이 지역에 있어서 안심할 수 있습니까?	①	②	③	④	⑤
(10)	귀하께서는 이 지역에 대해 소속감을 느끼십니까?	①	②	③	④	⑤
(11)	귀하께서는 이 지역사회를 개선하기 위해, 주위 사람들과 함께 무엇인가 하려고 생각하고 계십니까?	①	②	③	④	⑤
(12)	귀하께서는 이 지역에서 사는 것에 만족하십니까?	①	②	③	④	⑤
(13)	귀하께서는 선거 시 반드시 투표하십니까?	①	②	③	④	⑤
(14)	귀하께서는 이 지역의 이웃들이 소중하다고 생각하십니까?	①	②	③	④	⑤

2. 귀하께서는 다음과 같은 지역 모임이나 조직에 참여하고 계십니까?
(예. 봉사활동, 청년회, 노인회, 부인회, 학부모회, 스포츠 동아리, 자치회, 반상회, 소방단, 종교조직)

☐ ①예 (2-1번으로) ☐② 아니오 (3번으로)

2-1. 위의 조직에서 활동한 기간은 얼마입니까? 약 ________ 개월

3. 살고 있는 지역(마을)에 가족 이외에 가까이 지내는 사람이 몇 명입니까? 약 ________ 명

4. 현재 함께 살고 있는 동거인의 수는 몇 명입니까? ________ 명

5. 현재 함께 살고 있는 동거인은 누구입니까? (중복가능)

☐ ① 부모님 ☐② 자녀 ☐③ 친척 ☐④ 지인 ☐⑤ 기타( )

코로나바이러스감염증-19 (COVID-19)

※ 다음은 작년 초(2020년 1월) 이후 코로나19 유행과 관련된 질문입니다.

6. 귀하 또는 주변(가족 또는 이웃)에 코로나19 확진을 경험하신 적이 있습니까?		
□ ① 있다	□ ② 없다	□③ 모름

7. 자가격리를 해보신 적이 있습니까?		
□ ① 있다	□ ② 없다	□③ 모름

8. 원주시의 코로나19 대응능력이 적절하다고 생각하십니까?	
□ ① 매우 적절하다<br>□ ② 적절하다<br>□ ③ 보통	□ ④ 적절하지 않다<br>□ ⑤ 전혀 적절하지 않다

9. 코로나19 예방을 위하여 원주시에서 어떤 활동이 필요하다고 생각하십니까?<br>(거리두기 지침 마련, 백신 접종, 시민과의 위기소통, 방역물자 지원 등)
활동 ① ____
활동 ② ____
활동 ③ ____

10. 귀하는 백신을 어느 정도 신뢰하십니까?				
□ ① 매우 신뢰함	□② 신뢰함	□③ 보통	□④ 신뢰하지 않음	□⑤ 매우 신뢰하지 않음

11. 코로나19 백신을 접종받은 경험이 있습니까?<br>(*백신: 화이자, 아스트라제네카 등 모든 종류 포함)		
□ ① 있다 (▶ 46번으로)	□ ② 없다 (▶ 45-1 로)	□ ③ 기억나지 않음 (▶ 45-1 로)

11-1. 코로나19 백신을 접종받을 의향이 있습니까?		
□ ① 무조건 받을 것이다	□ ② 아마도 받을 것이다	□ ③ 아마도 받지 않을 것이다
□ ④ 절대 받지 않을 것이다	□ ⑤ 아직 모르겠다	

12. 보건소, 병원, 선별진료소 등에서 코로나19 검사를 받은 경험이 있습니까?		
□ ① 있다	□ ② 없다	□ ③ 기억나지 않음

일반적 특성

13. 귀하의 성별은?	
□① 남성	□② 여성

14. 귀하의 출생년도는?
______ 년

15. 현재 귀하의 키는 얼마입니까?
키: ______ cm

16. 현재 귀하의 몸무게는 어느정도이신지요?
몸무게: ___________ kg

17. 귀하께서 종사하고 있는 직종은 다음 중 무엇에 해당합니까?	
□① 공무원<br>□② 농업, 임업종사자<br>□③ 자영업, 서비스업<br>□④ 전문직(의사, 변호사, 교사 등)	□⑤ 학생, 재수생<br>□⑥ 회사원<br>□⑦ 주부<br>□⑧ 무직<br>□⑨ 기타 (_________________________)

18. 이자・임대수입, 외부 생활보조 정책 등을 모두 합한 귀댁의 월평균 가구 총 소득은 얼마입니까?	
□① 100만원 미만<br>□② 100~199만원<br>□③ 200~299만원	□④ 300~399만원<br>□⑤ 400~499만원<br>□⑥ 500만원 이상

19. 귀댁의 거주 형태는 어떻습니까?	
□① 맨션, 아파트<br>□② 단독주택<br>□③ 원룸	□④ 기숙사<br>□⑤ 기타 ( )

20. 귀하께서 원주시에 거주하신 기간은 얼마입니까?
____ 년 ____ 개월

이상. 끝.

참여해주셔서 감사합니다.
