# Supplementary material for: Influencing factor of COVID-19 vaccination trust and hesitancy in Wonju city, South Korea
Source: PLoS One. 2022 Nov 14;17(11):e0277016. doi: 10.1371/journal.pone.0277016 (PMC9662712; doi:10.1371/journal.pone.0277016)
Supplement: Attachment — Submitted filename: PONE-D-21-29174_R2_reviewer Review.pdf [file pone.0277016.s005.pdf]

# PLOS ONE

## Association between social capital and COVID-19 vaccine trust and hesitancy

--Manuscript Draft--

| | |
|---|---|
| **Manuscript Number:** | PONE-D-21-29174R2 |
| **Article Type:** | Research Article |
| **Full Title:** | Association between social capital and COVID-19 vaccine trust and hesitancy |
| **Short Title:** | Social capital and COVID-19 vaccine trust and hesitancy |
| **Corresponding Author:** | Eun Woo Nam<br>Yonsei University College of Health Sciences<br>Wonju, KOREA, REPUBLIC OF |
| **Keywords:** | COVID-19; social capital; COVID-19 Vaccine trust; COVID-19 vaccine hesitancy; South Korea |
| **Abstract:** | Social capital (SC) has been documented to effectively reduce the spread of diseases, including COVID-19; however, research pertaining to SC and COVID-19 vaccination in Korea is lacking. This cross-sectional study conducted in the city of Wonju, Gangwon Province, Korea (n =1,096) examined the differences in COVID-19 vaccine trust and hesitancy considering individual characteristics and investigated the effects of SC on COVID-19 vaccine trust and hesitancy. SC was measured based on 14 items pertaining to social trust, network, and norms. Responses regarding COVID-19 screening history, vaccine trust, and vaccine hesitancy were also assessed. SC scores did not differ between sexes, but differed significantly according to age and household income; thus, adults aged 70–79 years had the highest SC scores, and mean SC score increased significantly with income. COVID-19 vaccine trust differed significantly according to age, average household income, social organization involvement, and SC score. COVID-19 vaccine hesitancy differed significantly with age, SC score, and COVID-19 screening history. In univariate logistic regression, age, average household income, social organization involvement, and SC score were found to be significant predictors of vaccine trust; in multivariable analysis, however, the identified predictors were age and SC. In particular, people with an SC score ≥50 were 2.642 times more likely to trust COVID-19 vaccines than those with lower scores. In multivariable analysis, age and SC were significant predictors of vaccine hesitancy. In particular, people with an SC score ≥50 were 1.424 times more likely not to be hesitant about receiving COVID-19 vaccines than people with lower scores. These results indicate that prioritizing policies to increase SC and trust in the government could boost the COVID-19 vaccination rate. |
| **Order of Authors:** | Hocheol Lee |
| | Eun Bi Noh |
| | Ji Eon Kim |
| | Juyeon Oh |
| | Eun Woo Nam |
| **Response to Reviewers:** | Dear Reviewers,<br><br>First, we are very appreciated for your helpful review. I discussed with all authors for your comments. And we revised/corrected the context as all manuscript.<br>We attached the two manuscripts, 1) clean manuscript, 2) tracked manuscript. In the ' 2) track manuscript' file, our responses/revisions are highlighted using tracked system on word.<br>Thank you for review in advance.<br>Prof. Eun Woo Nam. |
| **Additional Information:** | |
| **Question** | **Response** |
| **Financial Disclosure** | This research was supported by the Basic Science Research Program through the |

Powered by Editorial Manager® and ProduXion Manager® from Aries Systems Corporation

| | |
|---|---|
| Enter a financial disclosure statement that describes the sources of funding for the work included in this submission. Review the submission guidelines for detailed requirements. View published research articles from *PLOS ONE* for specific examples.<br><br>This statement is required for submission and **will appear in the published article** if the submission is accepted. Please make sure it is accurate.<br><br>**Unfunded studies**<br>Enter: *The author(s) received no specific funding for this work.*<br><br>**Funded studies**<br>Enter a statement with the following details:<br>• Initials of the authors who received each award<br>• Grant numbers awarded to each author<br>• The full name of each funder<br>• URL of each funder website<br>• Did the sponsors or funders play any role in the study design, data collection and analysis, decision to publish, or preparation of the manuscript?<br>• **NO** - Include this sentence at the end of your statement: *The funders had no role in study design, data collection and analysis, decision to publish, or preparation of the manuscript.*<br>• **YES** - Specify the role(s) played.<br><br>* typeset | National Research Foundation of Korea (NRF) funded by the Ministry of Education (NRF-2021R1C1C2005464). The funders had no role in study design, data collection and analysis, decision to publish, or preparation of the manuscript. |
| **Competing Interests**<br><br>Use the instructions below to enter a competing interest statement for this submission. On behalf of all authors, disclose any competing interests that could be perceived to bias this work—acknowledging all financial support and any other relevant financial or non-financial competing interests.<br><br>This statement is required for submission and **will appear in the published article** if | The authors have declared that no competing interests exist. |

| | |
|---|---|
| the submission is accepted. Please make sure it is accurate and that any funding sources listed in your Funding Information later in the submission form are also declared in your Financial Disclosure statement.<br><br>View published research articles from *PLOS ONE* for specific examples.<br><br>**NO authors have competing interests**<br><br>Enter: *The authors have declared that no competing interests exist.*<br><br>**Authors with competing interests**<br><br>Enter competing interest details beginning with this statement:<br><br>*I have read the journal's policy and the authors of this manuscript have the following competing interests: [insert competing interests here]*<br><br>* typeset | |
| **Ethics Statement**<br><br>Enter an ethics statement for this submission. This statement is required if the study involved:<br><br>• Human participants<br>• Human specimens or tissue<br>• Vertebrate animals or cephalopods<br>• Vertebrate embryos or tissues<br>• Field research<br><br>Write "N/A" if the submission does not require an ethics statement.<br><br>General guidance is provided below. Consult the submission guidelines for detailed instructions. **Make sure that all information entered here is included in the Methods section of the manuscript.** | Ethical approval was received from the Yonsei University Committee (REC 1041849-202104-SB-063-02). |

| | |
|---|---|
| **Format for specific study types**<br><br>**Human Subject Research (involving human participants and/or tissue)**<br>• Give the name of the institutional review board or ethics committee that approved the study<br>• Include the approval number and/or a statement indicating approval of this research<br>• Indicate the form of consent obtained (written/oral) or the reason that consent was not obtained (e.g. the data were analyzed anonymously)<br><br>**Animal Research (involving vertebrate animals, embryos or tissues)**<br>• Provide the name of the Institutional Animal Care and Use Committee (IACUC) or other relevant ethics board that reviewed the study protocol, and indicate whether they approved this research or granted a formal waiver of ethical approval<br>• Include an approval number if one was obtained<br>• If the study involved *non-human primates*, add *additional details* about animal welfare and steps taken to ameliorate suffering<br>• If anesthesia, euthanasia, or any kind of animal sacrifice is part of the study, include briefly which substances and/or methods were applied<br><br>**Field Research**<br><br>Include the following details if this study involves the collection of plant, animal, or other materials from a natural setting:<br>• Field permit number<br>• Name of the institution or relevant body that granted permission | |
| **Data Availability**<br><br>Authors are required to make all data underlying the findings described fully available, without restriction, and from the time of publication. PLOS allows rare exceptions to address legal and ethical concerns. See the PLOS Data Policy and FAQ for detailed information. | No - some restrictions will apply |

| | |
|---|---|
| A Data Availability Statement describing where the data can be found is required at submission. Your answers to this question constitute the Data Availability Statement and **will be published in the article**, if accepted.<br><br>**Important:** Stating 'data available on request from the author' is not sufficient. If your data are only available upon request, select 'No' for the first question and explain your exceptional situation in the text box.<br><br>Do the authors confirm that all data underlying the findings described in their manuscript are fully available without restriction? | |
| **Describe where the data may be found in full sentences. If you are copying our sample text, replace any instances of XXX with the appropriate details.**<br><br>• If the data are **held or will be held in a public repository**, include URLs, accession numbers or DOIs. If this information will only be available after acceptance, indicate this by ticking the box below. For example: *All XXX files are available from the XXX database (accession number(s) XXX, XXX.).*<br>• If the data are all contained **within the manuscript and/or Supporting Information files**, enter the following: *All relevant data are within the manuscript and its Supporting Information files.*<br>• If neither of these applies but you are able to provide **details of access elsewhere**, with or without limitations, please do so. For example:<br><br>*Data cannot be shared publicly because of [XXX]. Data are available from the XXX Institutional Data Access / Ethics Committee (contact via XXX) for researchers who meet the criteria for access to confidential data.*<br><br>*The data underlying the results presented in the study are available from (include the name of the third party* | Data cannot be shared without permission by Wonju city hall(Healthy City team) publicly, South Korea. Data are available from the Wonju city hall Institutional Data Access(contact via Yeon-seok Jin, jys12076@korea.kr) / Ethics Committee (contact via yuwirb@yonsei.ac.kr/https://e-irb.yonsei.ac.kr/index.htm?) for researchers who meet the criteria for access to confidential data. |

Powered by Editorial Manager® and ProduXion Manager® from Aries Systems Corporation

| | |
|---|---|
| *and contact information or URL).*<br>• This text is appropriate if the data are owned by a third party and authors do not have permission to share the data.<br><br>* typeset | |
| Additional data availability information: | |

Powered by Editorial Manager® and ProduXion Manager® from Aries Systems Corporation

Manuscript

Click here to access/download;Manuscript;02-1. Clean_3nd_Manuscripts_Sep_05.docx



# Association between social capital and COVID-19 vaccine trust and hesitancy

Short title: Social capital and COVID-19 vaccine trust and hesitancy

Hocheol Lee[1¶], Eun Bi Noh[1,2], Ji Eon Kim[1,2], Juyeon Oh[3], Eun Woo Nam[1,2*]

[1] Yonsei Global Health Center, Yonsei University, Wonju, Gangwon-do, Republic of Korea

[2] Department of Health Administration, Yonsei University Graduate School, Wonju, Gangwon-do, Republic of Korea

[3] Department of Information Statistics, Yonsei University, Wonju, Gangwon-do, Republic of Korea

* Corresponding author

E-mail: ewnam@yonsei.ac.kr (EWN)

# Abstract

Social capital (SC) has been documented to effectively reduce the spread of diseases, including COVID-19; however, research pertaining to SC and COVID-19 vaccination in Korea is lacking. This cross-sectional study conducted in the city of Wonju, Gangwon Province, Korea (n =1,096) examined the differences in COVID-19 vaccine trust and hesitancy considering individual characteristics and investigated the effects of SC on COVID-19 vaccine trust and hesitancy. SC was measured based on 14 items pertaining to social trust, network, and norms. Responses regarding COVID-19 screening history, vaccine trust, and vaccine hesitancy were also assessed. SC scores did not differ between sexes, but differed significantly according to age and household income; thus, adults aged 70–79 years had the highest SC scores, and mean SC score increased significantly with income. COVID-19 vaccine trust differed significantly according to age, average household income, social organization involvement, and SC score. COVID-19 vaccine hesitancy differed significantly with age, SC score, and COVID-19 screening history. In univariate logistic regression, age, average household income, social organization involvement, and SC score were found to be significant predictors of vaccine trust; in multivariable analysis, however, the identified predictors were age and SC. In particular, people with an SC score ≥50 were 2.642 times more likely to trust COVID-19 vaccines than those with lower scores. In multivariable analysis, age and SC were significant predictors of vaccine hesitancy. In particular, people with an SC score ≥50 were 1.424 times more likely not to be hesitant about receiving COVID-19 vaccines than people with lower scores. These results indicate that prioritizing policies to increase SC and trust in the government could boost the COVID-19 vaccination rate.

Keywords: COVID-19; Social Capital; COVID-19 Vaccine trust; COVID-19 Vaccine Hesitancy; South Korea

# Introduction

Since the first case of coronavirus disease 2019 (COVID-19) was reported in Korea on January 19, 2020, there have been a total of 225,481 cases and 2,167 deaths as of August 16, 2021 [1]. Following the World Health Organization (WHO) vaccination guidelines, Korea began COVID-19 vaccine rollout in February 2021—starting from healthcare workers and moving on to older adults, epidemiology personnel, high-risk groups, and the general population—with a plan to achieve herd immunity by completely vaccinating 70% of the population by November 2021 [2].

In the Core Capacity Workbook for International Health Regulations, the World Health Organization (WHO) proposed eight core capacities for the control and prevention of the spread of infectious diseases. Furthermore, it emphasized that governments should be equipped with adequate vaccine transport infrastructure, healthcare facilities, risk communication and monitoring systems, finances, and social capital (SC) to recommend COVID-19 vaccination [3].

In the 1980s, SC was defined as "shared values in relation to connections and networks among individuals." It particularly encompasses social activities, trust, norms, and attachment [4,5] and has been consistently documented as strongly associated with individuals' physical and mental health [6]. Even during the COVID-19 pandemic, SC has been fund to effectively lower the associated direct and indirect health threats. Previous studies have reported that regions with higher SC responded more proactively to the spread of COVID-19 and showed reduced spread of the disease [7,8]. In particular, groups with high trust in the government, a factor of SC, strictly adhered to COVID-19 guidelines for the good of the social network

(community) and limited their daily movements in compliance with the government mandate [9].

The Korean government has initiated its COVID-19 vaccine rollout with the goal of achieving herd immunity by the second half of 2021. To this end, the government has striven to boost public trust in COVID-19 vaccines and encourage vaccination. While some studies have reported that SC positively impacts the decision to get vaccinated against COVID-19, research or evidence pertaining to SC and COVID-19 vaccination in Korea is lacking.

This study aimed to identify the effects of SC on COVID-19 vaccine trust and hesitancy, with two specific objectives: 1) to examine the differences in COVID-19 vaccine trust and hesitancy according to various participant characteristics and 2) to investigate the effects of SC on COVID-19 vaccine trust and hesitancy.

# Methods

## Study design

This cross-sectional study was conducted in the city of Wonju, Gangwon Province, Republic of Korea. The city of Wonju is divided into 14 *dong* (urban areas) and 9 *myeon* (rural areas). In 2020, its population was 357,710, of whom 290,157 were adults.

## Study instrument

The study instrument was developed with a focus on SC, COVID-19 responses, and general

characteristics. First, SC was measured based on 14 items pertaining to social trust (4 items), social network (5 items), and social norms (5 items), with each item rated on a 5-point Likert-type scale. Second, COVID-19-related responses were assessed using items developed in the context of the study region and based on the annual Community Health Survey conducted by Statistics Korea. The questions asked were regarding COVID-19 screening history, COVID-19 vaccine trust, and COVID-19 vaccine hesitancy. The general characteristics assessed were sex, age, average household income, and area of residence. Ages were divided into 20s, 30s, 40s, 50s, and 60s. House income was classified as <1 million, 1–1.99 million, 2–2.99 million, 3–3.99 million, 4–4.99 million, and >5 million. This was based on the classification table of the Community Health Survey in Korea.

## Data collection

Adult residents of Wonju aged 20 years or older were enrolled in this study. The study sample was extracted via probability proportional to size sampling to ensure that it was representative of the population. In Step 1, the sample size was allocated proportionately to age and population for each of the 23 urban and rural areas of Wonju. In Step 2, the final sample was randomly selected from the allocated sample by each region. Using Raosoft, the minimum sample size required for a confidence level of 95% and significance of 5% was calculated at 1,197.

For the questionnaire survey, 18 enumerators were hired and trained from April 26 to May 3, 2022. A pilot survey was conducted in four regions on May 5, and the main survey was conducted from May 7 to May 17. A total of 1,248 participants completed the survey. After excluding those with careless responses (e.g. censored respondents) and those who withdrew from the survey, a total of 1,096 participants were included. We obtained informed consent in

written form from all the respondents. The respondents were informed of their right to refuse to answer questions that were sent to the participants.

## Statistical analysis

We used the following statistical techniques to analyze the effects of SC on COVID-19 vaccine trust and hesitancy. First, SC according to each characteristic was visualized using box plots. Second, the differences in COVID-19 vaccine trust and hesitancy according to general characteristics, social organization involvement, SC score, and COVID-19 infection history were analyzed using Pearson's chi-square tests. Third, predictors of COVID-19 vaccine trust and hesitancy were identified using binary logistic regression. All regression coefficients, odds ratios, *t*-values, and *p*-values were examined to determine the predictability of each factor of COVID-19 vaccine trust and hesitancy.

All statistical analyses were conducted using STATA 15, and the data were visualized using the R-4.11 software.

## Ethical considerations

All components of this survey were approved by the institutional review board (IRB) of Yonsei University (IRB document number: 1041849-202104-SB-063-02).

## Results

## Participant characteristics

There were more urban (72.4%) than rural dwellers (27.6%) and more men (53.5%) than women (46.5%) among the study participants. The most common age group was 20–29 years (20.4%). A total of 31.0% of the participants were involved in a social organization, and 60.4% had an SC score of <50. A total of 23.3% of the participants had undergone COVID-19 screening, 30.2% trusted COVID-19 vaccines, and 62.2% were willing to be vaccinated against COVID-19 (Table 1).

**Table 1. Characteristics of respondents (n=1,096)**

| | N | % |
|---|---|---|
| Area of residence | | |
| Urban | 793 | 72.4 |
| Rural | 303 | 27.6 |
| Sex | | |
| Male | 586 | 53.5 |
| Female | 510 | 46.5 |
| Age | | |
| 20–29 years | 224 | 20.4 |
| 30–39 years | 212 | 19.3 |
| 40–49 years | 206 | 18.8 |
| 50–59 years | 213 | 19.4 |
| ≥ 60years | 241 | 22.0 |
| Average household income | | |

| | | |
|---|---|---|
| ≤ 1 million KRW | 113 | 103 |
| 1.00–1.99 million KRW | 136 | 12.4 |
| 2.00–2.99 million KRW | 202 | 18.4 |
| 3.00–3.99 million KRW | 233 | 21.3 |
| 4.00–4.99 million KRW | 166 | 15.1 |
| ≥ 5 million KRW | 246 | 22.4 |
| Social organization involvement | | |
| Yes | 340 | 31.0 |
| No | 756 | 69.0 |
| Social capital score | | |
| <50 | 662 | 60.4 |
| ≥50 | 434 | 39.6 |
| COVID-19 screening | | |
| Yes | 255 | 23.3 |
| No | 841 | 76.7 |
| COVID-19 vaccine trust | | |
| Yes | 331 | 30.2 |
| No | 765 | 69.8 |
| COVID-19 vaccine hesitancy | | |
| Not hesitant | 682 | 62.2 |
| Hesitant | 414 | 37.8 |

KRW: Korean won

The visualization of SC scores based on participant characteristics using box plots (Fig. 1) showed that SC scores did not differ between sexes, but did differ significantly according to age, with adults aged 70–79 years having the highest SC scores ($p<0.000$). SC score also differed according to household income, with the mean SC score increasing significantly with increasing income ($p<0.000$). While the mean SC score was lower in the socially involved group, there were many outliers. Moreover, SC scores differed according to COVID-19 vaccine

trust, with the group that did not trust COVID-19 vaccines having higher SC scores (*p*<0.000).

**Fig 1. Social capital by participant characteristics**

## COVID-19 trust and hesitancy by participant characteristics

Next, the differences in COVID-19 vaccine trust and hesitancy according to participant characteristics were analyzed using Pearson's chi-squared tests (Table 2). COVID-19 vaccine trust differed significantly according to age (*p*<0.000), average household income (*p*=0.040), social organization involvement (*p*=0.001), and SC score (*p*<0.000). COVID-19 vaccine hesitancy differed significantly according to age (*p*<.000), SC score (*p*=0.003), and COVID-19 screening history (*p*=0.039).

**Table 2. Differences in COVID-19 vaccine trust and hesitancy**

| | COVID-19 vaccine trust | | $\chi^2$ (*p*) | COVID-19 vaccine hesitancy | | $\chi^2$ (*p*) |
|---|---|---|---|---|---|---|
| | Yes | No | | Yes | No | |
| Area of residence | | | | | | |
| Urban | 299 (28.9%) | 564 (71.1%) | 2.382 (0.123) | 499 (63.1%) | 292 (36.9%) | 0.675 (0.411) |
| Rural | 102 (33.7%) | 201 (63.3%) | | 183 (60.4%) | 120 (39.6%) | |
| Sex | | | | | | |

| | | | | | | |
|---|---|---|---|---|---|---|
| Male | 189 (32.3%) | 397 (67.7%) | 2.515 (0.113) | 363 (61.9%) | 223 (38.1%) | 0.084 (0.772) |
| Female | 142 (27.8%) | 368 (72.2%) | | 319 (62.8%) | 189 (37.2%) | |
| Age | | | | | | |
| 20–29 years | 39 (17.4%) | 185 (82.6%) | 58.881. (<0.000) | 117 (52.5%) | 106 (47.5%) | 35.972 (<0.000) |
| 30–39 years | 46 (21.7%) | 166 (78.3%) | | 111 (52.4%) | 101 (47.6%) | |
| 40–49 years | 59 (28.6%) | 147 (71.4%) | | 144 (69.9%) | 62 (30.1%) | |
| 50–59 years | 74 (34.7%) | 139 (65.3%) | | 142 (66.7%) | 71 (33.3%) | |
| ≥60 years | 113 (46.9%) | 128 (53.1%) | | 168 (70.0%) | 72 (30.0%) | |
| Average household income | | | | | | |
| ≤ 1 million KRW | 49 (43.4%) | 64 (56.6%) | 11.666 (0.040) | 69 (61.1%) | 44 (38.9%) | 3.876 (0.567) |
| 1.00–1.99 million KRW | 40 (29.4%) | 96 (70.6%) | | 78 (57.4%) | 58 (42.6%) | |
| 2.00–2.99 million KRW | 52 (25.7%) | 150 (74.3%) | | 125 (61.9%) | 77 (38.1%) | |
| 3.00–3.99 million KRW | 66 (28.3%) | 167 (71.7%) | | 143 (61.4%) | 90 (38.6%) | |
| 4.00–4.99 million KRW | 49 (29.5%) | 117 (70.5%) | | 112 (67.9%) | 53 (32.1%) | |

| | | | | | | |
|---|---|---|---|---|---|---|
| ≥ 5 million KRW | 75 (30.5%) | 171 (69.5%) | | 155 (63.3%) | 90 (32.1%) | |
| Social organization involvement | | | | | | |
| Yes | 127 (27.0%) | 213 (73.0%) | 11.961 (0.001) | 221 (61.1%) | 119 (38.9%) | 1.487 (0.223) |
| No | 204 (37.4%) | 552 (62.6%) | | 461 (65.0%) | 293 (35.0%) | |
| Social capital score | | | | | | |
| <50 | 142 (21.5%) | 520 (78.5%) | 60.727 (<0.000) | 388 (56.9%) | 272 (41.2%) | 8.941 (0.003) |
| ≥50 | 189 (43.5%) | 245 (56.5%) | | 294 (67.7%) | 140 (32.3%) | |
| COVID-19 screening | | | | | | |
| Yes | 71 (30.9%) | 184 (69.1%) | 0.876 (0.349) | 145 (64.0%) | 110 (36.0%) | 4.249 (0.039) |
| No | 260 (27.8%) | 581 (72.2%) | | 537 (56.9%) | 302 (43.1%) | |

KRW: Korean won

## Predictors of COVID-19 vaccine trust and hesitancy

Binary logistic regression analysis was performed to identify the predictors of COVID-19 vaccine trust and hesitancy (Table 3). In the univariate analysis of vaccine trust, age, average household income, social organization involvement, and SC score were identified as significant predictors. In the multivariable analysis, age and SC score were identified as significant predictors. In particular, people with an SC score of ≥50 were 2.660

times more likely to trust COVID-19 vaccines than those with an SC score of <50 (*p*<0.001).

In multivariable logistic regression analysis of predictors of COVID-19 hesitancy, age and SC were identified as significant predictors. In particular, people with an SC score of ≥50 were 1.400 times more likely not to be hesitant about receiving COVID-19 vaccines than people with an SC score of <50 (*p*<0.01).

**Table 3. Results of logistic regression for predictors of COVID-19 vaccine trust and hesitancy**

| Variable | COVID-19 vaccine trust | | | | COVID-19 vaccine hesitancy | | | |
|---|---|---|---|---|---|---|---|---|
| | cOR | 95% CI | aOR | 95% CI | cOR | 95% CI | aOR | 95% CI |
| Area of residence | | | | | | | | |
| Urban | Ref. | | Ref. | | Ref | | Ref. | |
| Rural | 1.250 | 0.941–1.659 | 1.185 | 0.874–1.606 | 0.892 | 0.680–1.171 | 0.858 | 0.648–1.135 |
| Sex | | | | | | | | |
| Male | Ref. | | Ref. | | Ref | | Ref. | |
| Female | .811 | 0.625–1.051 | .808 | 0.612–1.066 | 1.037 | 0.811–1.325 | 1.018 | 0.790–1.312 |
| Age | | | | | | | | |
| 20–29 years | Ref. | | Ref. | | Ref | | Ref. | |
| 30–39 years | 1.314 | 0.817–2.114 | 1.369 | 0.835–2.246 | 0.982 | 0.683–1.451 | 0.976 | 0.663–1.438 |
| 40–49 years | 1.904** | 1.203–3.012 | 2.000** | 1.230–3.230 | 2.104*** | 1.415–3.130 | 2.049** | 1.359–3.090 |

| | | | | | | | | |
|---|---|---|---|---|---|---|---|---|
| 50–59 years | 2.525*** | 1.617–3.944 | 2.386*** | 1.488–3.8263.819 | 1.812** | 1.230–2.670 | 1.706* | 1.138–2.558 |
| ≥60 years | 4.188*** | 2.729–6.425 | 3.710**** | 2.323–5.923 | 2.114*** | 1.444–3.095 | 2.225*** | 1.469–3.370 |
| Average household income | | | | | | | | |
| ≤1 million KRW | Ref. | | Ref. | | Ref | | Ref. | |
| 1.00–1.99 million KRW | .544* | 0.322–0.919 | 0.643 | 0.366–1.128 | 0.858 | 0.516–1.426 | 0.573 | 0.573–1.636 |
| 2.00–2.99 million KRW | .453** | 0.278–0.737 | 0.628 | 0..370–1.067 | 1.035 | 0.645–1.661 | 1.337 | 0.812–2.202 |
| 3.00–3.99 million KRW | .516** | 0.323–0.825 | 0.15 | 0.432–1.237 | 1.013 | 0.639–1.607 | 1.217 | 0.746–1.984 |
| 4.00–4.99 million KRW | .547** | 0.332–0.902 | 0.687 | 0.410–1.149 | 1.348 | 0.818–2.221 | 1.553 | 0.911–2.647 |
| ≥5 million KRW | .573** | 0.361–0.908 | 0.841 | 0.410–0.245 | 1.098 | 0.694–1.737 | 1.383 | 0.845–2.262 |
| Social organization involvement | | | | | | | | |
| Yes | Ref. | | Ref. | | Ref | | Ref. | |
| No | 1.613*** | 1.229–2.118 | 1.065 | 0.790–1.4207 | 1.180 | 0.904–1.541 | .965 | 0.724–1.287 |
| COVID-19 screening | | | | | | | | |

| | | | | | | | | |
|---|---|---|---|---|---|---|---|---|
| No | Ref. | | Ref. | | Ref | | Ref. | |
| Yes | .862 | 0.632–1.176 | 1.006 | 0.723–1.402 | 0.741* | 0.557–0.986 | 0.803 | 0.598–1.819 |
| Social capital score | | | | | | | | |
| <50 | Ref. | | Ref. | | Ref | | Ref. | |
| ≥50 | 2.825*** | 2.166–3.684 | 2.660*** | 2.018–3.504 | 1.472** | 1.142–1.898 | 1.400** | 1.077–1.819 |
| Hosmer–Lemeshow | | | .222 | | | | | .112 |

*$p$<0.05, **$p$<0.01, ***$p$<0.001

KRW: Korean won, aOR: adjusted odds ratio

# Discussion

This study aimed to analyze the effects of SC on COVID-19 vaccine trust and hesitancy. Our results showed that SC scores did not differ between sexes but differed significantly according to age and increased with increasing household income. COVID-19 vaccine trust differed significantly according to age, average household income, social organization involvement, and SC score. COVID-19 vaccine hesitancy differed significantly according to age, SC score, and COVID-19 screening history. In univariate logistic regression, age, average household income, social organization involvement, and SC score were found to be significant predictors of vaccine trust; in multivariable analysis, the identified predictors were age and SC. In multivariable analysis, age and SC were significant predictors of vaccine hesitancy.

Regarding the demographic characteristics of the participants, 72.4% were urban dwellers, which was similar to the reported percentage of urban dwellers (76.6%) for the city of Wonju [10], an urban-rural complex city. A total of 31.0% of the participants were involved in a social organization, which was higher than the rate (23.0%) reported in a previous study [11]. In our study, 30.2% of the participants stated that they trusted COVID-19 vaccines, which was lower than the global rate of vaccine trust (42.2%) [12]. Regarding COVID-19 vaccine hesitancy, 62.2% of the participants said they were not hesitant about being vaccinated, which was low considering that the average level of willingness to be vaccinated against COVID-19 worldwide is higher than 70.0% [13,14]. The reasons for this low willingness may include the continuous decline of the Korean government's approval ratings, limitations of risk communication, and spread of negative views due to the COVID-19 infodemic via the Internet and media [15]. A strong belief in inaccurate information about COVID-19 has been found to be influenced more by social media than the news [16], and considering that social media usage escalated by 87% during the COVID-19 pandemic compared to that in the pre-COVID-19 period, the younger generation—comprising the predominant users of social media—is likely to be vulnerable to the infodemic [17].

SC has been reported to influence health, well-being, social support, sociability, and social standing; it has a particularly greater impact on older adults [18–20]. In our study sample, SC increased with age and increasing social involvement. One of the reasons for higher SC among older individuals is that older people engage in fewer economic activities, and social welfare policies are mostly focused on recommending and encouraging the social involvement of older adults [21].

The first objective of this study was to analyze the differences in COVID-19 vaccine trust and hesitancy according to the various characteristics of the study participants. COVID-

19 vaccine trust was found to differ significantly according to age, household income, social involvement, and SC score, while COVID-19 vaccine hesitancy differed significantly according to age, SC score, and COVID-19 screening history. We confirmed that COVID-19 vaccine trust and acceptance increased significantly with advancing age, which is consistent with previous findings [22]. In particular, a recent telephonic survey of 1,200 people in Hong Kong showed that 42.2% of the respondents trusted COVID-19 vaccines and were willing to be vaccinated. By age, the COVID-19 vaccine acceptance rate was below 10% in the age group 16–54 years, but it increased with advancing age to 21.7% among those aged ≥55 years and 53.5% among those aged ≥65 years [22]. Increased COVID-19 vaccine trust and acceptance with advancing age is associated with the channels through which individuals acquire information and also the frequency of receiving information. Young individuals are frequently exposed to potentially inaccurate information through social media and are likely to encounter rumors and misinformation. In contrast, older adults generally obtain information about COVID-19 vaccines through public mass media, such as news outlets, and they are thus given relatively more credible information, which has probably contributed to boosting their COVID-19 vaccine trust [16].

The second objective of this study was to identify the predictors of COVID-19 vaccine trust and hesitancy. Age and SC score were identified as predictors of COVID-19 vaccine trust and hesitancy. People with higher SC scores showed higher levels of COVID-19 vaccine trust and acceptance. SC comprises four dimensions—civic engagement, trust in the government, social belonging, and social trust—and a high SC score indicates that the individual trusts both the social community and the government and is actively involved in activities as a member of the society [23,24]. Previous studies have reported that SC is effective in provoking behaviors that reduce COVID-19-related health risk [9], and that regions with greater SC deal with

infectious diseases more proactively and thus have fewer cases in the community [7,8]. SC-oriented public health responses can complement the limitations of top-down policies, and it is important to boost social solidarity and trust to achieve the public health goal of battling COVID-19 [25]. Thus, SC is expected to increase the COVID-19 vaccination rate and positively contribute to the efforts of communities to halt the spread of the disease. In particular, a previous study has reported that civic engagement and trust in the government had positive effects on COVID-19 responses, while trust in and a sense of belonging in affiliated groups had a negative effect on COVID-19 responses [26]. Another study reported that among the various elements of SC, low trust in institutions hindered COVID-19 response measures such as physical distancing [27].

Moreover, societies with high social trust may actually be more vulnerable to fake news about the severity of COVID-19, fake treatments, and criticisms against policies such as physical distancing [28].

This study has a few limitations. First, due to the cross-sectional study design, we could not identify the causative factors in a time-series analysis from 2020, when the index case of COVID-19 occurred in Korea. Second, although the city of Wonju, where this study was conducted, has a population of 300,000, the findings cannot be expected to be nationally representative. Subsequent studies should recruit participants from other and more diverse regions to ensure the generalizability of the findings in Korea.

# Conclusion

This study confirmed that SC is a predictor of COVID-19 vaccine trust and hesitancy,

and that people with higher SC have greater trust in COVID-19 vaccines and are more willing to be vaccinated. The Korean government chose herd immunity as a key strategy for controlling COVID-19 and has implemented COVID-19 vaccination plans accordingly. Our results suggest that the government should strive to increase SC to boost the COVID-19 vaccination rate, which is crucial in pursuing long-term COVID-19 response measures in response to the prolonged pandemic. In particular, the government should prioritize policies to increase social involvement and trust in the government. To this end, the Korean government will need to transparently provide reliable COVID-19 information through various channels such as the media and the Internet through COVID-19 experts and celebrities to increase its credibility.

**Acknowledgments**

We express our gratitude to everyone working to overcome COVID-19 in South Korea and throughout the world. Furthermore, we express our gratitude to the Healthy City team, Wonju City Hall.

# References

1. Available from: http://ncov.mohw.go.kr/en/.
2. Korea Centers for Disease Control & Prevention. Free vaccination for all citizens of COVID-19 to return to daily life. Cheongju: Korea Centers for Disease Control & Prevention; 2021.

3. Felter C. A guide to global COVID-19 vaccine efforts. 2021 Jun 30. Council on Foreign Relations. Available from: https://www.jstor.org/stable/resrep33363

4. Coleman JS. Social Capital in the Creation of human capital. Am J Sociol. 1988;94: S95–S120. doi: 10.1086/228943.

5. Putnam RD. Bowling alone: the collapse and revival of American community. New York: Simon and Schuster; 2000.

6. Rodgers J, Valuev AV, Hswen Y, Subramanian SV. Social capital and physical health: an updated review of the literature for 2007–2018. Soc Sci Med. 2019;236: 112360. doi: 10.1016/j.socscimed.2019.112360.

7. Borgonovi F, Andrieu E. Bowling together by bowling alone: social capital and COVID-19. Soc Sci Med. 2020;265:113501. Doi: 10.1016/j.socscimed.2020.113501

8. Makridis CA, Wu C. How social capital helps communities weather the COVID-19 pandemic. PLOS ONE. 2021;16(9):e0258021. https://doi.org/10.1371/journal.pone.0258021 9. Bian Y, Miao X, Lu X, Ma X, Guo X. The emergence of a COVID-19 related social capital: the case of China. Int J Sociol. 2020;50: 419–433. doi: 10.1080/00207659.2020.1802141.

10. Wonju Statistical. Information. 2020. Available from: https://stat.wonju.go.kr/board/board.php?b_id=res_stat.

11. Yıldızer G, Bilgin E, Korur EN, Novak D, Demirhan G. The association of various social capital indicators and physical activity participation among Turkish adolescents. J Sport Health Sci. 2018;7: 27-33. doi: 10.1016/j.jshs.2017.10.008.

12. Willis DE, Andersen JA, Bryant-Moore K, Selig JP, Long CR, Felix HC, et al. COVID-19 vaccine hesitancy: race/ethnicity, trust, and fear. Clin Transl Sci. 2021;14:2200–2207.

doi: 10.1111/cts.13077.

13. Sallam M. COVID-19 vaccine hesitancy worldwide: a concise systematic review of vaccine acceptance rates. Vaccines. 2021;9: 160. doi: 10.3390/vaccines9020160.
14. Trent M, Seale H, Chughtai AA, Salmon D, MacIntyre CR. Trust in government, intention to vaccinate and COVID-19 vaccine hesitancy: A comparative survey of five large cities in the United States, United Kingdom, and Australia. Vaccine. 2022;40: 2498–2505. doi: 10.1016/j.vaccine.2021.06.048.
15. Lee H, Noh EB, Choi SH, Zhao B, Nam EW. Determining public opinion of the COVID-19 pandemic in South Korea and Japan: social network mining on twitter. Healthc Inform Res. 2020;26: 335-343. doi: 10.4258/hir.2020.26.4.335.
16. Nielsen RK, Fletcher R, Newman N, Brennen JS, Howard PN. Navigating the 'infodemic': how people in six countries access and rate news and information about coronavirus. Reuters Institute; 2020.
17. Sharma P, Raje M, Savant S, Savant R. When infodemic overtakes Covid-19 in spreading far and wide. Ann Rom Soc Cell Biol. 2021: 4721-4730.
18. Cramm JM, Van Dijk HM, Nieboer AP. The importance of neighborhood social cohesion and social capital for the well being of older adults in the community. Gerontologist. 2013;53: 142-152. doi: 10.1093/geront/gns052.
19. Lin N, Erickson BH, editors. Social capital: an international research program. Oxford University Press; 2010.
20. Nieminen T, Prättälä R, Martelin T, Härkänen T, Hyyppä MT, Alanen E, et al. Social capital, health behaviours and health: a population-based associational study. BMC Public Health. 2013;13: 613. doi: 10.1186/1471-2458-13-613.
21. Gray A. The social capital of older people. Ageing Soc. 2009;29: 5-31. doi:

10.1017/S0144686X08007617.

22. Wong MCS, Wong ELY, Huang J, Cheung AWL, Law K, Chong MKC, et al. Acceptance of the COVID-19 vaccine based on the health belief model: A population-based survey in Hong Kong. Vaccine. 2021;39: 1148-1156. doi: 10.1016/j.vaccine.2020.12.083.

23. Pearson AL. The (social distanced) circle of family, friends, and allies: how COVID-19 is re-shaping social capital and new opportunities for research. In: Andrews GJ, Crooks, VA, Pearce JR, Messina JP, editors. COVID-19 and similar futures. Global perspectives on health geography. Springer, Cham; 2021. p. 139-145.

24. Jiang N, Lou VWQ, Lu N. Does social capital influence preferences for aging in place? Evidence from urban China. Aging Ment Health. 2018;22: 405-411. doi: 10.1080/13607863.2016.1249455.

25. Wong ASY, Kohler JC. Social capital and public health: responding to the COVID-19 pandemic. Global Health. 2020;16: 88. doi: 10.1186/s12992-020-00615-x.

26. Villalonga-Olives E, Kawachi I. The dark side of social capital: A systematic review of the negative health effects of social capital. Soc Sci Med. 2017;194: 105–127. doi: 10.1016/j.socscimed.2017.10.020.

27. Dezecache G, Frith CD, Deroy O. Pandemics and the great evolutionary mismatch. Curr Biol. 2020;30: R417–R419. doi: 10.1016/j.cub.2020.04.010.

28. Zmerli S. Social capital and norms of citizenship: an ambiguous relationship? Am Behav Sci. 2010;53: 657–676. doi: 10.1177/0002764209350829.

Click here to access/download;Figure;revised_Fig1_june 29.png

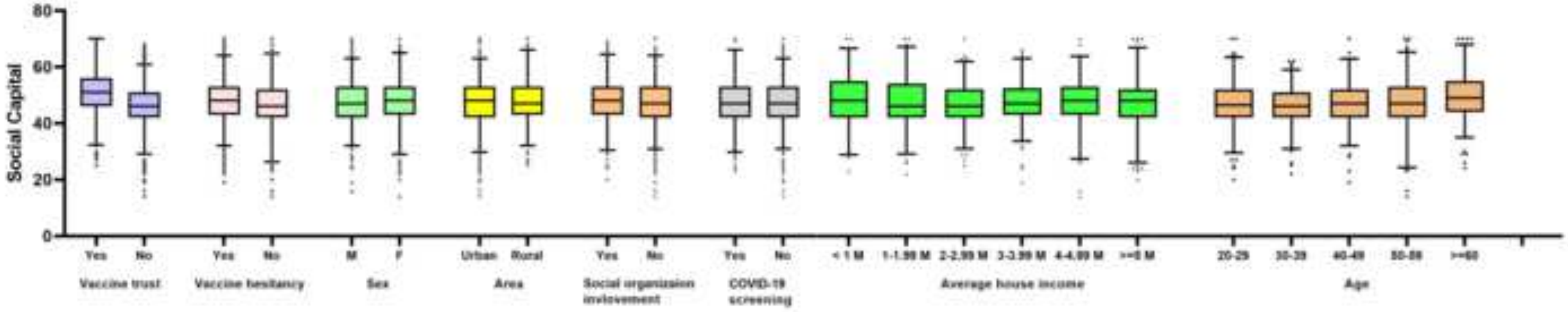

(KOR) questionnaire

Click here to access/download
**Supporting Information**
(KOR) Questionnaire.pdf

(ENG) questionnaire

Click here to access/download
**Supporting Information**
(ENG) Questionnaire.pdf

Revised Manuscript with Track Changes



# Association between social capital and COVID-19 vaccine trust and hesitancy

Short title: Social capital and COVID-19 vaccine trust and hesitancy

Hocheol Lee[1¶], Eun Bi Noh[1,2], Ji Eon Kim[1,2], Juyeon Oh[3], Eun Woo Nam[1,2*]

[1] Yonsei Global Health Center, Yonsei University, Wonju, Gangwon-do, Republic of Korea

[2] Department of Health Administration, Yonsei University Graduate School, Wonju, Gangwon-do, Republic of Korea

[3] Department of Information Statistics, Yonsei University, Wonju, Gangwon-do, Republic of Korea

* Corresponding author

E-mail: ewnam@yonsei.ac.kr (EWN)

# Abstract

~~The Korean government aims to completely vaccinate 70% of the population against coronavirus diseases 2019 (COVID-19) by November 2021.~~ Social capital (SC) has been documented to effectively reduce the spread of diseases, including COVID-19; however, research pertaining to SC and COVID-19 vaccination in Korea is lacking. This cross-sectional study conducted in the city of Wonju, Gangwon Province, Korea (n =1,096) examined~~aimed to examine~~ the differences in COVID-19 vaccine trust and hesitancy ~~according to~~considering ~~subject~~ individual characteristics and investigated the effects of SC on COVID-19 vaccine trust and hesitancy. SC was measured based on 14 items pertaining to social trust, ~~social~~ network, and ~~social~~ norms. Responses regarding COVID-19 screening history, vaccine trust, and vaccine hesitancy were also assessed. SC scores did not differ between sexes, but differed significantly according to age and household income; thus, ~~e~~ adults aged 70–79 years had the highest SC scores, and mean SC score increased significantly with ~~increasing~~ income. COVID-19 vaccine trust differed significantly according to age, average household income, social organization involvement, and SC score. COVID-19 vaccine hesitancy differed significantly ~~according to~~with age, SC score, and COVID-19 screening history. In univariate logistic regression, age, average household income, social organization involvement, and SC score were found to be significant predictors of vaccine trust; in multivariable analysis, however, the identified predictors were age and SC. In particular, people with an SC score ≥50 ~~or higher~~ were 2.642 times more likely to trust COVID-19 vaccines than those with lower scores. In multivariable analysis, age and SC were significant predictors of vaccine hesitancy. In particular, people with an SC score ≥50 ~~or higher~~ were 1.424 times more likely not to be hesitant about receiving COVID-

19 vaccines than people with lower scores. These results indicate that prioritizing policies to increase SC and trust in the government could boost the COVID-19 vaccination rate.

Keywords: COVID-19; Social Capital; COVID-19 Vaccine trust; COVID-19 Vaccine Hesitancy; South Korea

# Introduction

Since the first case of coronavirus disease 2019 (COVID-19) was reported in Korea on January 19, 2020, there have been a total of 225,481 cases and 2,167 deaths~~,~~ as of August 16, 2021 [1]. ~~With reference to~~Following the World Health Organization (WHO) vaccination guidelines, Korea began COVID-19 vaccine rollout in February 2021—starting from health~~-~~care workers and moving on to older adults, epidemiology personnel, high-risk groups, and the general population—with a plan to achieve herd immunity by completely vaccinating 70% of the population by November 2021 [2].

In the Core Capacity Workbook for International Health Regulations, the World Health Organization (WHO) ~~proposes~~ proposed eight core capacities for the control and prevention of the spread of infectious diseases. Furthermore, ~~the WHO~~it emphasized that governments should be equipped with adequate vaccine transport infrastructure, health~~-~~care facilities, risk communication and monitoring systems, finances, and social capital (SC) to recommend COVID-19 vaccination [3].

In the 1980s, SC was defined as "shared values in relation to connections and networks among individuals."~~.~~ It particularly encompasses social activities, trust, norms, and attachment [4,5]~~,~~ and has been consistently documented ~~to be~~as strongly associated with individuals' physical and mental health [6]~~;~~. ~~e~~Even during the COVID-19 pandemic, SC ~~could~~ has been fund to effectively lower the associated direct and indirect health threats. Previous studies have reported that regions with higher SC responded more proactively to the spread of COVID-19 and showed reduced spread of the disease [7,8]. In particular, groups with high trust in the government, ~~which is one of the~~ a factor~~s~~ of SC, strictly adhered to COVID-19 guidelines for

the good of the social network (community) and limited their daily movements in compliance with the government mandate [9].

The Korean government has initiated its COVID-19 vaccine rollout with the goal of achieving herd immunity by the second half of 2021. To this end, the government has ~~strives~~ striven to boost public trust in COVID-19 vaccines and encourage vaccination. While some studies have reported that SC ~~has a~~ positively impacts ~~on~~ the decision to get vaccinated against COVID-19, research or evidence pertaining to SC and COVID-19 vaccination in Korea is lacking.

~~The aim of this~~This study ~~was~~ aimed to identify the effects of SC on COVID-19 vaccine trust and hesitancy~~. We have~~ , with two specific objectives: 1) to examine the differences in COVID-19 vaccine trust and hesitancy according to various ~~subject~~ participant characteristics~~,~~ and 2) to investigate the effects of SC on COVID-19 vaccine trust and hesitancy.

# Methods

## Study design

This cross-sectional study was conducted in the city of Wonju, Gangwon Province, Republic of Korea. The city of Wonju is divided into 14 *dong* (urban areas) and 9 *myeon* (rural areas). ~~and had~~In 2020, its ~~a~~ population ~~of~~ was 357,710 ~~in 2020~~, of whom 290,157 were adults. ~~Adult residents of Wonju aged 20 years or older were enrolled in this study.~~

## Study instrument

The study instrument was developed with a focus on SC, COVID-19 responses, and general characteristics. First, SC was measured ~~on the basis of~~based on 14 items pertaining to social trust (4 items), social network (5 items), and social norms (5 items), with each item rated on a ~~five~~5-point Likert-type scale. Second, COVID-19-related responses were assessed using items developed in the context of the study region and based on the annual Community Health Survey conducted by Statistics Korea. The questions asked were regarding COVID-19 screening history, COVID-19 vaccine trust, and COVID-19 vaccine hesitancy. The general characteristics assessed were sex, age, average household income, and area of residence. Ages were divided into 20~~'~~s, 30~~'~~s, 40~~'~~s, 50~~'~~s, and 60~~'~~s. House income was classified as ~~less than~~ <1 million, 1–~~-~~1.99 million, 2–~~-~~2.99 million ~~or more~~, 3–~~-~~3.99 million ~~or more~~, 4–~~-~~4.99 million ~~or more~~, and >5 million ~~or more~~. This was based on the classification table of the Community Health Survey in Korea.

## Data collection

Adult residents of Wonju aged 20 years or older were enrolled in this study. The study sample was extracted via probability proportional to size sampling to ensure that it was representative of the population. In S~~s~~tep 1, the sample size was allocated proportionately to age and population for each of the 23 urban and rural areas of Wonju~~*myeon* and *dong*~~. In S~~s~~tep 2, the final sample was randomly selected from the allocated sample by each region. Using Raosoft, the minimum sample size required for a confidence level of 95% and significance of 5% was calculated ~~to be~~at 1,197.

For the questionnaire survey, 18 enumerators were hired and trained from April 26 to May 3, 2022. A pilot survey was conducted in four regions on May 5, and the main survey was conducted from May 7 to May 17. A total of 1,248 participants completed the survey~~;~~. ~~a~~After

excluding those with careless responses (e.g. censored respondents) and those who withdrew from the survey, a total of 1,096 participants were included in the analysis. We obtained informed consent in written form from all the respondents. It must be noted that special notifications on tThe respondents' were informed of their rights to refuse to answer questions that were sent to the participants.

## Statistical analysis

We used the following statistical techniques to analyze the effects of SC on COVID-19 vaccine trust and hesitancy. First, SC according to each characteristic was visualized using box plots. Second, the differences in COVID-19 vaccine trust and hesitancy according to general characteristics, social organization involvement, SC score, and COVID-19 infection history were analyzed using Pearson's chi-square tests. Third, predictors of COVID-19 vaccine trust and hesitancy were identified using binary logistic regression. All regression coefficients, odds ratios, *t*-values, and *p*-values were examined to determine the predictability of each factor of COVID-19 vaccine trust and hesitancy.

All statistical analyses were conducted using STATA 15, and the data were visualized using the R-4.11 software.

## Ethical considerations

All components of this survey were approved by the institutional review board (IRB) of Yonsei University (IRB document number: 1041849-202104-SB-063-02). Written informed consent was obtained from all respondents prior to data collection. In particular, we drew

attention to respondents' right to refuse the survey request on the first page of the online survey form.

# Results

## Participant characteristics

There were more urban (72.4%) than rural dwellers (27.6%) and more men (53.5%) than women (46.5%) among the study participants. The most common age group was 20–29 years (20.4%). A total of 31.0% of the participants were involved in a social organization, and 60.4% of the participants had an SC score of less than≤ 50. A total of 23.3% of the participants had undergone COVID-19 screening, 30.2% trusted COVID-19 vaccines, and 62.2% were willing to be vaccinated against COVID-19 (Table 1).

**Table 1. Characteristics of respondents (n=1,096)**

| | N | % |
|---|---|---|
| Area of residence | | |
| Urban | 793 | 72.4 |
| Rural | 303 | 27.6 |

| | | |
|---|---|---|
| Sex | | |
| Male | 586 | 53.5 |
| Female | 510 | 46.5 |
| Age | | |
| 20–29 years | 224 | 20.4 |
| 30–39 years | 212 | 19.3 |
| 40–49 years | 206 | 18.8 |
| 50–59 years | 213 | 19.4 |
| ≥ 60years | 241 | 22.0 |
| Average household income | | |
| ≤ 1 million KRW | 113 | 103 |
| 1.00–1.99 million KRW | 136 | 12.4 |
| 2.00–2.99 million KRW | 202 | 18.4 |
| 3.00–3.99 million KRW | 233 | 21.3 |
| 4.00–4.99 million KRW | 166 | 15.1 |
| ≥ 5 million KRW | 246 | 22.4 |
| Social organization involvement | | |
| Yes | 340 | 31.0 |
| No | 756 | 69.0 |
| Social capital score | | |
| <50 | 662 | 60.4 |
| ≥50 | 434 | 39.6 |
| COVID-19 screening | | |
| Yes | 255 | 23.3 |
| No | 841 | 76.7 |
| COVID-19 vaccine trust | | |
| Yes | 331 | 30.2 |
| No | 765 | 69.8 |
| COVID-19 vaccine hesitancy | | |

| | | |
|---|---|---|
| Not hesitant | 682 | 62.2 |
| Hesitant | 414 | 37.8 |

KRW: Korean won

The v~~V~~isualization of SC scores based on participant characteristics using box plots (Fig. 1) showed that SC scores did not differ between sexes, but did differ significantly according to age, with adults aged 70–79 years having the highest SC scores (*p*<0.000). SC score also differed according to household income, with the mean SC score increasing significantly with increasing income (*p*<0.000). While the mean SC score was lower in the socially involved group, there were many outliers. Moreover, SC scores differed according to COVID-19 vaccine trust, with the group that did not trust COVID-19 vaccines having higher SC scores (*p*<0.000).

**Fig 1. Social capital by participant characteristics**

## COVID-19 trust and hesitancy by participant characteristics

Next, the differences in COVID-19 vaccine trust and hesitancy according to participant characteristics were analyzed using ~~the~~ Pearson's chi-squared tests (Table 2). COVID-19 vaccine trust differed significantly according to age (*p*<0.000), average household income (*p*=0.040), social organization involvement (*p*=0.001), and SC score (*p*<0.000). COVID-19 vaccine hesitancy differed significantly according to age (*p*<.000), SC score (*p*=0.003), and COVID-19 screening history (*p*=0.039).

**Table 2. Differences in COVID-19 vaccine trust and hesitancy**

| | COVID-19 vaccine trust | | $\chi^2$ ($p$) | COVID-19 vaccine hesitancy | | $\chi^2$ ($p$) |
|---|---|---|---|---|---|---|
| | Yes | No | | Yes | No | |
| Area of residence | | | | | | |
| Urban | 299 (28.9%) | 564 (71.1%) | 2.382 (0.123) | 499 (63.1%) | 292 (36.9%) | 0.675 (0.411) |
| Rural | 102 (33.7%) | 201 (63.3%) | | 183 (60.4%) | 120 (39.6%) | |
| Sex | | | | | | |
| Male | 189 (32.3%) | 397 (67.7%) | 2.515 (0.113) | 363 (61.9%) | 223 (38.1%) | 0.084 (0.772) |
| Female | 142 (27.8%) | 368 (72.2%) | | 319 (62.8%) | 189 (37.2%) | |
| Age | | | | | | |
| 20–29 years | 39 (17.4%) | 185 (82.6%) | 58.881. (<0.000) | 117 (52.5%) | 106 (47.5%) | 35.972 (<0.000) |
| 30–39 years | 46 (21.7%) | 166 (78.3%) | | 111 (52.4%) | 101 (47.6%) | |
| 40–49 years | 59 (28.6%) | 147 (71.4%) | | 144 (69.9%) | 62 (30.1%) | |
| 50–59 years | 74 (34.7%) | 139 (65.3%) | | 142 (66.7%) | 71 (33.3%) | |
| ≥60 years | 113 (46.9%) | 128 (53.1%) | | 168 (70.0%) | 72 (30.0%) | |
| Average household income | | | | | | |

| | | | | | | |
|---|---|---|---|---|---|---|
| ≤ 1 million KRW | 49 (43.4%) | 64 (56.6%) | 11.666 (0.040) | 69 (61.1%) | 44 (38.9%) | 3.876 (0.567) |
| 1.00–1.99 million KRW | 40 (29.4%) | 96 (70.6%) | | 78 (57.4%) | 58 (42.6%) | |
| 2.00–2.99 million KRW | 52 (25.7%) | 150 (74.3%) | | 125 (61.9%) | 77 (38.1%) | |
| 3.00–3.99 million KRW | 66 (28.3%) | 167 (71.7%) | | 143 (61.4%) | 90 (38.6%) | |
| 4.00–4.99 million KRW | 49 (29.5%) | 117 (70.5%) | | 112 (67.9%) | 53 (32.1%) | |
| ≥ 5 million KRW | 75 (30.5%) | 171 (69.5%) | | 155 (63.3%) | 90 (32.1%) | |
| Social organization involvement | | | | | | |
| Yes | 127 (27.0%) | 213 (73.0%) | 11.961 (0.001) | 221 (61.1%) | 119 (38.9%) | 1.487 (0.223) |
| No | 204 (37.4%) | 552 (62.6%) | | 461 (65.0%) | 293 (35.0%) | |
| Social capital score | | | | | | |
| <50 | 142 (21.5%) | 520 (78.5%) | 60.727 (<0.000) | 388 (56.9%) | 272 (41.2%) | 8.941 (0.003) |
| ≥50 | 189 (43.5%) | 245 (56.5%) | | 294 (67.7%) | 140 (32.3%) | |
| COVID-19 screening | | | | | | |
| Yes | 71 (30.9%) | 184 (69.1%) | 0.876 (0.349) | 145 (64.0%) | 110 (36.0%) | 4.249 (0.039) |
| No | 260 (27.8%) | 581 (72.2%) | | 537 (56.9%) | 302 (43.1%) | |

KRW: Korean won

## Predictors of COVID-19 vaccine trust and hesitancy

Binary logistic regression analysis was performed to identify the predictors of COVID-19 vaccine trust and hesitancy (Table 3). In the univariate analysis of vaccine trust, age, average household income, social organization involvement, and SC score were identified as significant predictors. In the multivariable analysis, age and SC score were identified as significant predictors. In particular, people with an SC score of ≥50 ~~or higher~~ were 2.660 times more likely to trust COVID-19 vaccines than those with an SC score ~~less than~~ of <50 (*p*<0.001).

In multivariable logistic regression analysis of predictors of COVID-19 hesitancy, age and SC were identified as significant predictors. In particular, people with an SC score of ≥50 ~~or higher~~ were 1.400 times more likely not to be hesitant about receiving COVID-19 vaccines than people with an SC score of <~~less than~~ 50 (*p*<0.01).

**Table 3. Results of logistic regression for predictors of COVID-19 vaccine trust and hesitancy**

| Variable | COVID-19 vaccine trust | | | | COVID-19 vaccine hesitancy | | | |
|---|---|---|---|---|---|---|---|---|
| | cOR | 95% CI | aOR | 95% CI | cOR | 95% CI | aOR | 95% CI |
| Area of residence | | | | | | | | |
| Urban | Ref. | | Ref. | | Ref | | Ref. | |
| Rural | 1.250 | 0.941–1.659 | 1.185 | 0.874–1.606 | 0.892 | 0.680–1.171 | 0.858 | 0.648–1.135 |

| | | | | | | | | |
|---|---|---|---|---|---|---|---|---|
| Sex | | | | | | | | |
| Male | Ref. | | Ref. | | Ref | | Ref. | |
| Female | .811 | 0.625–1.051 | .808 | 0.612–1.066 | 1.037 | 0.811–1.325 | 1.018 | 0.790–1.312 |
| Age | | | | | | | | |
| 20–29 years | Ref. | | Ref. | | Ref | | Ref. | |
| 30–39 years | 1.314 | 0.817–2.114 | 1.369 | 0.835–2.246 | 0.982 | 0.683–1.451 | 0.976 | 0.663–1.438 |
| 40–49 years | 1.904** | 1.203–3.012 | 2.000** | 1.230–3.230 | 2.104*** | 1.415–3.130 | 2.049** | 1.359–3.090 |
| 50–59 years | 2.525*** | 1.617–3.944 | 2.386*** | 1.488–3.8263.819 | 1.812** | 1.230–2.670 | 1.706* | 1.138–2.558 |
| ≥60 years | 4.188*** | 2.729–6.425 | 3.710**** | 2.323–5.923 | 2.114*** | 1.444–3.095 | 2.225*** | 1.469–3.370 |
| Average household income | | | | | | | | |
| ≤—1 million KRW | Ref. | | Ref. | | Ref | | Ref. | |
| 1.00–1.99 million KRW | .544* | 0.322–0.919 | 0.643 | 0.366–1.128 | 0.858 | 0.516–1.426 | 0.573 | 0.573–1.636 |
| 2.00–2.99 million KRW | .453** | 0.278–0.737 | 0.628 | 0..370–1.067 | 1.035 | 0.645–1.661 | 1.337 | 0.812–2.202 |
| 3.00–3.99 million KRW | .516** | 0.323–0.825 | 0.15 | 0.432–1.237 | 1.013 | 0.639–1.607 | 1.217 | 0.746–1.984 |
| 4.00–4.99 million KRW | .547** | 0.332–0.902 | 0.687 | 0.410–1.149 | 1.348 | 0.818–2.221 | 1.553 | 0.911–2.647 |

| | | | | | | | | |
|---|---|---|---|---|---|---|---|---|
| ≥5 million KRW | .573** | 0.361–0.908 | 0.841 | 0.410–0.245 | 1.098 | 0.694–1.737 | 1.383 | 0.845–2.262 |
| Social organization involvement | | | | | | | | |
| Yes | Ref. | | Ref. | | Ref | | Ref. | |
| No | 1.613*** | 1.229–2.118 | 1.065 | 0.790–1.4207 | 1.180 | 0.904–1.541 | .965 | 0.724–1.287 |
| COVID-19 screening | | | | | | | | |
| No | Ref. | | Ref. | | Ref | | Ref. | |
| Yes | .862 | 0.632–1.176 | 1.006 | 0.723–1.402 | 0.741* | 0.557–0.986 | 0.803 | 0.598–1.819 |
| Social capital score | | | | | | | | |
| <50 | Ref. | | Ref. | | Ref | | Ref. | |
| ≥50 | 2.825*** | 2.166–3.684 | 2.660*** | 2.018–3.504 | 1.472** | 1.142–1.898 | 1.400** | 1.077–1.819 |
| Hosmer–Lemeshow | | | .222 | | | | | .112 |

*$p$<0.05, **$p$<0.01, ***$p$<0.001

KRW: Korean won, aOR: adjusted odds ratio

# Discussion

T~~he aim of t~~his study ~~was~~ aimed to analyze the effects of SC on COVID-19 vaccine trust and hesitancy. Our results showed that SC scores did not differ between sexes~~,~~ but differed significantly according to age and increased with increasing household income.

COVID-19 vaccine trust differed significantly according to age, average household income, social organization involvement, and SC score. COVID-19 vaccine hesitancy differed significantly according to age, SC score, and COVID-19 screening history. In univariate logistic regression, age, average household income, social organization involvement, and SC score were found to be significant predictors of vaccine trust; in multivariable analysis, the identified predictors were age and SC. In multivariable analysis, age and SC were significant predictors of vaccine hesitancy.

Regarding the demographic characteristics of the participants, 72.4% were urban dwellers, which was similar to the reported percentage of urban dwellers (76.6%) for the city of Wonju [10], ~~which is~~ an urban-rural complex city. A total of 31.0% of the participants were involved in a social organization, which ~~is~~ was higher than the rate (23.0%) reported in a previous study [11]. In our study, 30.2% of the participants stated that they trusted COVID-19 vaccines, which ~~is~~ was lower than the global rate of vaccine trust (42.2%) [12]. Regarding COVID-19 vaccine hesitancy, 62.2% of the participants said they were not hesitant about being vaccinated, which ~~is~~ was low considering that the average level of willingness to be vaccinated against COVID-19 worldwide is higher than 70.0% [13,14]. The reasons for this low willingness may include the continuous decline of the Korean government’s approval ratings, limitations of risk communication, and spread of negative views due to the COVID-19 infodemic via the Internet and media [15]. ~~It has been documented that a~~A strong belief in inaccurate information about COVID-19 ~~is~~ has been found to be influenced more by social media than the news [16], and considering that social media usage escalated by 87% during the COVID-19 pandemic compared to that in the pre-COVID-19 period, the younger generation—comprising the predominant users of social media—is likely to be vulnerable to the infodemic [17].

SC has been reported to influence health, well-being, social support, sociability, and social standing~~,~~; ~~and~~ it has a particularly greater impact on older adults [18–20]. In our study sample, SC increased with ~~advancing~~ age and increasing social involvement. One of the reasons for higher SC among older individuals is that older people engage in fewer economic activities, and social welfare policies are mostly focused on recommending and encouraging the social involvement of older adults [21].

The first objective of this study was to analyze the differences in COVID-19 vaccine trust and hesitancy according to the various characteristics of the study participants. COVID-19 vaccine trust was found to differ significantly according to age, household income, social involvement, and SC score, while COVID-19 vaccine hesitancy differed significantly according to age, SC score, and COVID-19 screening history. We confirmed that COVID-19 vaccine trust and acceptance increased significantly with advancing age, which is consistent with previous findings [22]. In particular, a recent telephonic survey of 1,200 people in Hong Kong showed that 42.2% of the respondents trusted COVID-19 vaccines and were willing to be vaccinated. By age, the COVID-19 vaccine acceptance rate was below 10% in the age group 16–54 years~~ age group~~, but it increased with advancing age to 21.7% among those aged ≥~~-~~55 years and 53.5% among those aged ≥~~-~~65 years [22]. Increased COVID-19 vaccine trust and acceptance with advancing age is associated with the channels through which individuals acquire information and ~~the~~ also the frequency of receiving information. Young individuals are frequently exposed to potentially inaccurate information through social media and are likely to ~~be exposed to~~encounter rumors and misinformation. ~~On the other hand~~In contrast, older adults generally obtain information about COVID-19 vaccines through public mass media, such as news outlets, and they are thus given relatively more credible information, which has probably contributed to boosting their COVID-19 vaccine trust [16].

The second objective of this study was to identify the predictors of COVID-19 vaccine trust and hesitancy. Age and SC score were identified as predictors of COVID-19 vaccine trust and hesitancy. People with higher SC scores showed higher levels of COVID-19 vaccine trust and acceptance. SC comprises four dimensions—civic engagement, trust in the government, social belonging, and social trust—and a high SC score indicates that the individual trusts both the social community and the government and is actively involved in activities as a member of the society [23,24]. Previous studies have reported that SC is effective in provoking behaviors that reduce COVID-19-related health risk [9], and that regions with greater SC deal with infectious diseases more proactively and thus have fewer cases in the community [~~25~~7,~~26~~8]. SC-oriented public health responses can complement the limitations of top-down policies, and it is important to boost social solidarity and trust ~~in order~~ to achieve the public health goal of battling COVID-19 [~~27~~25]. Thus, SC is expected to increase the COVID-19 vaccination rate and positively contribute to the efforts of communities to halt the spread of the disease. In particular, a previous study has reported that civic engagement and trust in the government had positive effects on COVID-19 responses, while trust in and a sense of belonging in affiliated groups had a negative effect on COVID-19 responses [~~28~~26]. Another study reported that among the various elements of SC, low trust in institutions hindered COVID-19 response measures such as physical distancing [~~29~~27].

Moreover, societies with high social trust may actually be more vulnerable to fake news about the severity of COVID-19, fake treatments, and criticisms against policies such as physical distancing [~~30~~28].

This study has a few limitations. First, due to the cross-sectional study design, we could not identify the causative factors in a time-series analysis from 2020, when the index case of COVID-19 occurred in Korea. Second, although the city of Wonju, where this study was

conducted, has a population of 300,000, the findings cannot be expected to be nationally representative. Subsequent studies should recruit ~~subjects~~ participants from other and more diverse regions to ensure the generalizability of the findings in Korea.

# Conclusion

This study confirmed that SC is a predictor of COVID-19 vaccine trust and hesitancy, and that people with higher SC have greater trust in COVID-19 vaccines and are more willing to be vaccinated. The Korean government chose herd immunity as a key strategy for controlling COVID-19 and has implemented COVID-19 vaccination plans accordingly. Our results suggest that the government should strive to increase SC to boost the COVID-19 vaccination rate, which is crucial ~~for~~ in pursuing long-term COVID-19 response measures in response to the prolonged pandemic. In particular, the government should prioritize policies to increase social involvement and trust in the government. To this end, the Korean government will need to transparently provide reliable COVID-19 information through various channels such as the media and the Internet through COVID-19 experts and celebrities to increase its credibility.

**Acknowledgments**

We express our gratitude to everyone working to overcome COVID-19 in South Korea and throughout the world. ~~And~~ Furthermore, we ~~appreciate~~ express our gratitude to ~~to~~ the Healthy City team, Wonju City ~~hall~~Hall.

# References


1. Available from: http://ncov.mohw.go.kr/en/.
2. Korea Centers for Disease Control & Prevention. Free ~~V~~vaccination for ~~A~~all ~~C~~citizens of COVID-19 to ~~R~~return to ~~D~~daily ~~L~~life. Cheongju: Korea Centers for Disease Control & Prevention; 2021.
3. Felter C~~laire~~. A ~~G~~guide to ~~G~~global COVID-19 ~~V~~vaccine ~~E~~efforts. 2021 Jun 30. Council on Foreign Relations. Available from: https://www.jstor.org/stable/resrep33363
4. Coleman JS. Social Capital in the Creation of human capital. Am J Sociol~~.~~. 1988;94: S95–S120. ~~DOI~~doi: 10.1086/228943.
5. Putnam R~~obert~~ D. ~~Simon and Schuster; New York, NY: 2000.~~ Bowling ~~A~~alone: the ~~C~~collapse and ~~R~~revival of American ~~C~~community. New York: Simon and Schuster; 2000.
6. Rodgers J, Valuev AV, Hswen Y, Subramanian SV. Social capital and physical health: an updated review of the literature for 2007–2018. Soc Sci Med~~.~~. 2019;236: 112360. doi: 10.1016/j.socscimed.2019.112360.
7. ~~Francesca~~ Borgonovi F, ~~Elodie~~ Andrieu E. Bowling together by bowling alone: social capital and ~~Covid~~COVID-19. ~~Covid Econ~~Soc Sci Med.~~,~~ 2020;~~17~~265:113501~~:73–96~~. Doi: 10.1016/j.socscimed.2020.113501
8. Makridis CA, Wu C. How social capital helps communities weather the COVID-19 pandemic. PLOS ONE. 2021;16(9):e0258021. https://doi.org/10.1371/journal.pone.0258021 ~~Makridis CA, Wu C. Ties that bind (and social distance): how social capital helps communities weather the COVID-19 pandemic;~~


**Formatted:** Font: (Default) Times New Roman, 12 pt
**Formatted:** Font: 12 pt
**Formatted:** Font: 12 pt, Not Italic
**Formatted:** Font: 12 pt
**Formatted:** Font: (Default) Times New Roman, 12 pt
**Formatted:** Font: 12 pt
**Formatted:** Font: (Default) Times New Roman, 12 pt, English (United States)
**Formatted:** Font: (Default) Times New Roman, 12 pt
**Formatted:** Font: 12 pt
**Formatted:** Font: 12 pt
**Formatted:** Font: (Default) Times New Roman, 12 pt
**Formatted:** Font: 12 pt
**Formatted:** Font: (Default) Times New Roman, 12 pt, English (United States)
**Formatted:** Font: (Default) Times New Roman, 12 pt
**Formatted:** Font: 12 pt
**Formatted:** Font: 12 pt
**Formatted:** Font: 12 pt
**Formatted:** Font: 12 pt
**Formatted:** Font: 12 pt
**Formatted:** Font: 12 pt
**Formatted:** Font: 12 pt
**Formatted:** Font: (Default) Times New Roman, 12 pt
**Formatted:** Font: 12 pt

~~2020.~~

9. Bian Y, Miao X, Lu X, Ma X, Guo X. The emergence of a COVID-19 related social capital: the case of China. Int J Sociol~~.~~. 2020;50~~(5)~~: 419–433. doi: 10.1080/00207659.2020.1802141.
10. Wonju Statistical. Information~~:~~. 2020. Available from: https://stat.wonju.go.kr/board/board.php?b_id=res_stat.
11. Yıldızer G, Bilgin E, Korur EN, Novak D, Demirhan G. The association of various social capital indicators and physical activity participation among Turkish adolescents. J Sport Health Sci~~.~~. 2018;7~~(1)~~: 27-33. ~~DOI~~doi: 10.1016/j.jshs.2017.10.008.
12. Willis D~~on~~ E, Andersen JA, Bryant-Moore K, Selig JP, Long CR, Felix HC, et al. COVID- 19 vaccine hesitancy: ~~R~~race/ethnicity, trust, and fear. Clin Transl Sci~~.~~. 2021;14:2200–2207. ~~DOI~~doi: 10.1111/cts.13077.
13. Sallam M~~alik~~. COVID-19 vaccine hesitancy worldwide: a concise systematic review of vaccine acceptance rates. Vaccines. 2021;9~~.2~~: 160. ~~DOI~~doi: 10.3390/vaccines9020160.
14. Trent M, Seale H, Chughtai AA, Salmon D, MacIntyre CR. Trust in government, intention to vaccinate and COVID-19 vaccine hesitancy: A comparative survey of five large cities in the United States, United Kingdom, and Australia. Vaccine. ~~2021~~2022;40: 2498–2505. ~~DOI~~doi: 10.1016/j.vaccine.2021.06.048.
15. Lee H, Noh EB, Choi SH, Zhao B, Nam EW. Determining public opinion of the COVID-19 pandemic in South Korea and Japan: social network mining on twitter. Healthc Inform Res~~.~~. 2020;26~~(4)~~: 335-343. ~~DOI~~doi: 10.4258/hir.2020.26.4.335.
16. Nielsen RK, Fletcher R, Newman N, Brennen JS, Howard PN. Navigating the ‘infodemic’: ~~H~~how people in six countries access and rate news and information about coronavirus. Reuters Institute; 2020.

**Formatted:** Font: (Default) Times New Roman, 12 pt
**Formatted:** Font: 12 pt
**Formatted:** Font: (Default) Times New Roman, 12 pt, English (United States)
**Formatted:** Font: (Default) Times New Roman, 12 pt
**Formatted:** Font: 12 pt
**Formatted:** Font: (Default) Times New Roman, 12 pt, English (United States)
**Formatted:** Font: (Default) Times New Roman, 12 pt
**Formatted:** Font: 12 pt
**Formatted:** Font: (Default) Times New Roman, 12 pt, English (United States)
**Formatted:** Font: (Default) Times New Roman, 12 pt
**Formatted:** Font: 12 pt
**Formatted:** Font: (Default) Times New Roman, 12 pt, English (United States)
**Formatted:** Font: (Default) Times New Roman, 12 pt
**Formatted:** Font: 12 pt
**Formatted:** Font: (Default) Times New Roman, 12 pt, English (United States)
**Formatted:** Font: (Default) Times New Roman, 12 pt
**Formatted:** Font: 12 pt
**Formatted:** Font: (Default) Times New Roman, 12 pt
**Formatted:** Font: (Default) Times New Roman, 12 pt, English (United States)
**Formatted:** Font: 12 pt
**Formatted:** Font: (Default) Times New Roman, 12 pt
**Formatted:** Font: 12 pt
**Formatted:** Font: (Default) Times New Roman, 12 pt, English (United States)
**Formatted:** Font: (Default) Times New Roman, 12 pt
**Formatted:** Font: 12 pt

17. Sharma P, Raje M, Savant S, Savant R. When ~~I~~infodemic ~~O~~overtakes Covid-19 in ~~S~~spreading ~~F~~far and ~~W~~wide. Ann Rom Soc Cell Biol~~.~~. 2021: 4721-4730.

18. Cramm JM, Van Dijk HM, Nieboer AP. The importance of neighborhood social cohesion and social capital for the well being of older adults in the community. Gerontologist. 2013;53~~(1)~~: 142-152. ~~DOI~~doi: 10.1093/geront/gns052.

19. Lin N, Erickson BH, ~~(Eds.~~editors~~)~~. Social capital: an international research program. Oxford University Press; 2010.

20. Nieminen T, Prättälä R, Martelin T, Härkänen T, Hyyppä MT, Alanen E, et al. Social capital, health behaviours and health: a population-based associational study. BMC Public Health. 2013;13~~(1)~~: 613. ~~DOI~~doi: 10.1186/1471-2458-13-613.

21. Gray A~~.~~. The social capital of older people. Ageing Soc~~.~~. 2009;29~~(1)~~: 5-31. ~~DOI~~doi: 10.1017/S0144686X08007617.

22. Wong MCS, Wong ELY, Huang J, Cheung AWL, Law K, Chong MKC, et al. Acceptance of the COVID-19 vaccine based on the health belief model: A population-based survey in Hong Kong. Vaccine. 2021;39~~(7)~~: 1148-1156. ~~DOI~~doi: 10.1016/j.vaccine.2020.12.083.

23. Pearson AL. The (~~S~~social ~~D~~distanced) ~~C~~circle of ~~F~~family, ~~F~~friends, and ~~A~~allies: ~~H~~how COVID-19 ~~I~~is ~~R~~re-shaping ~~S~~social ~~C~~capital and ~~N~~new ~~O~~opportunities for ~~R~~research. In: Andrews GJ, Crooks, VA, Pearce JR, Messina JP, editors. COVID-19 and similar futures. Global perspectives on health geography. Springer, Cham; 2021. p. 139-145.

24. Jiang N, Lou VWQ, Lu N. Does social capital influence preferences for aging in place? Evidence from urban China. Aging Ment Health. 2018;22~~(3)~~: 405-411. ~~DOI~~doi: 10.1080/13607863.2016.1249455.

~~25. rancesca Borgonovi, Elodie Andrieu Bowling together by bowling alone: social capital and Covid-19 Covid Econ. 2020;17: 73-96.~~


**Formatted:** Font: (Default) Times New Roman, 12 pt
**Formatted:** Font: 12 pt
**Formatted:** Font: (Default) Times New Roman, 12 pt
**Formatted:** Font: 12 pt
**Formatted:** Font: (Default) Times New Roman, 12 pt, English (United States)
**Formatted:** Font: (Default) Times New Roman, 12 pt
**Formatted:** Font: 12 pt
**Formatted:** Font: (Default) Times New Roman, 12 pt
**Formatted:** Font: 12 pt
**Formatted:** Font: (Default) Times New Roman, 12 pt, English (United States)
**Formatted:** Font: (Default) Times New Roman, 12 pt
**Formatted:** Font: 12 pt
**Formatted:** Font: (Default) Times New Roman, 12 pt, English (United States)
**Formatted:** Font: (Default) Times New Roman, 12 pt
**Formatted:** Font: 12 pt
**Formatted:** Font: (Default) Times New Roman, 12 pt, English (United States)
**Formatted:** Font: (Default) Times New Roman, 12 pt
**Formatted:** Font: 12 pt
**Formatted:** Font: (Default) Times New Roman, 12 pt
**Formatted:** Font: 12 pt
**Formatted:** Font: (Default) Times New Roman, 12 pt
**Formatted:** Font: 12 pt
**Formatted:** Font: (Default) Times New Roman, 12 pt, English (United States)
**Formatted:** Font: (Default) Times New Roman, 12 pt
**Formatted:** Font: 12 pt

~~26. Makridis CA, Wu C. Ties that bind (and social distance): how social capital helps communities weather the COVID-19 pandemic.~~

~~27~~25. Wong ASY, Kohler JC. Social capital and public health: responding to the COVID-19 pandemic. Global Health. 2020;16~~(1)~~: 88. ~~DOI~~doi: 10.1186/s12992-020-00615-x.

~~28~~26. Villalonga-Olives E, Kawachi I. The dark side of social capital: A systematic review of the negative health effects of social capital. Soc Sci Med~~.~~. 2017;~~Volume~~ 194: 105–127. ~~DOI~~doi: 10.1016/j.socscimed.2017.10.020.

~~29~~27. Dezecache G, Frith CD, Deroy O. Pandemics and the great evolutionary mismatch. Curr Biol~~.~~. 2020;30: R417–R419. ~~DOI~~doi: 10.1016/j.cub.2020.04.010.

~~30~~28. Zmerli S~~.~~. Social capital and norms of citizenship: an ambiguous relationship? Am Behav Sci~~.~~. 2010;53: 657–676. ~~DOI~~doi: 10.1177/0002764209350829.

**Formatted:** Font: (Default) Times New Roman, 12 pt
**Formatted:** Font: 12 pt
**Formatted:** Font: (Default) Times New Roman, 12 pt
**Formatted:** Font: (Default) Times New Roman, 12 pt
**Formatted:** Font: 12 pt
**Formatted:** Font: (Default) Times New Roman, 12 pt, English (United States)
**Formatted:** Font: (Default) Times New Roman, 12 pt
**Formatted:** Font: (Default) Times New Roman, 12 pt
**Formatted:** Font: 12 pt
**Formatted:** Font: (Default) Times New Roman, 12 pt
**Formatted:** Font: (Default) Times New Roman, 12 pt, English (United States)
**Formatted:** Font: 12 pt
**Formatted:** Font: (Default) Times New Roman, 12 pt
**Formatted:** Font: (Default) Times New Roman, 12 pt
**Formatted:** Font: 12 pt
**Formatted:** Font: (Default) Times New Roman, 12 pt, English (United States)
**Formatted:** Font: (Default) Times New Roman, 12 pt
**Formatted:** Font: (Default) Times New Roman, 12 pt
**Formatted:** Font: 12 pt
**Formatted:** Font: (Default) Times New Roman, 12 pt, English (United States)

**Comments to the Author**

**Authors**: First, we are very appreciated for your helpful review. I discussed with all authors for your comments. And we revised/corrected the context as all manuscript.

We attached the two manuscripts, 1) clean manuscript, 2) tracked manuscript. In the ' 2) track manuscript' file, our responses/revisions are highlighted using tracked system on word.

Thank you for review in advance.

Prof. Eun Woo Nam.

---

1. If the authors have adequately addressed your comments raised in a previous round of review and you feel that this manuscript is now acceptable for publication, you may indicate that here to bypass the "Comments to the Author" section, enter your conflict of interest statement in the "Confidential to Editor" section, and submit your "Accept" recommendation.

Reviewer #1: (No Response)

---

2. Is the manuscript technically sound, and do the data support the conclusions?

The manuscript must describe a technically sound piece of scientific research with data that supports the conclusions. Experiments must have been conducted rigorously, with appropriate controls, replication, and sample sizes. The conclusions must be drawn appropriately based on the data presented.

Reviewer #1: Partly

**Authors**: We corrected the context as a whole manuscript. Please review the attachment.

---

3. Has the statistical analysis been performed appropriately and rigorously?

Reviewer #1: N/A

---

4. Have the authors made all data underlying the findings in their manuscript fully available?

The PLOS Data policy requires authors to make all data underlying the findings described in their manuscript fully available without restriction, with rare exception (please refer to the Data Availability Statement in the manuscript PDF file). The data should be provided as part of the manuscript or its supporting information, or deposited to a public repository. For example, in addition to summary statistics, the data points behind means, medians and variance measures should be available. If there are restrictions on publicly sharing data—e.g. participant privacy or use of data from a third party—those must be specified.

Reviewer #1: No

**Authors**: Data cannot be shared without permission by Wonju city hall publicly, South Korea.

Data are available from the Wonju city hall Institutional Data Access / Ethics Committee (contact via Eun Woo Nam) for researchers who meet the criteria for access to confidential data.

---

5. Is the manuscript presented in an intelligible fashion and written in standard English?

PLOS ONE does not copyedit accepted manuscripts, so the language in submitted articles must be clear, correct, and unambiguous. Any typographical or grammatical errors should be corrected at revision, so please note any specific errors here.

Reviewer #1: No

**Authors**

1. We corrected the sentence, context as a whole manuscript. We also were followed the instructions available at https://journals.plos.org/plosone/s/submission-guidelines.

2. Note that I have added line numbers per journal guidelines: "Include page numbers and line numbers in the manuscript file. Use continuous line numbers (do not restart the numbering on each page)."

3. Note that I have added line numbers per journal guidelines: "Include page numbers and line numbers in the manuscript file. Use continuous line numbers (do not restart the numbering on each page)."

4. (Abstract) I have removed this sentence "The Korean government aims to completely ……" from the abstract as it gives little significance to the background of the study; you may reserve this for the main body. Until then, it's best to be direct in your abstract. The statement "Social capital (SC) has been documented to effectively reduce the spread of diseases, including COVID-19" is enough to introduce your study's aim.

5. We checked the all reference.

---

6. Review Comments to the Author

Please use the space provided to explain your answers to the questions above. You may also include additional comments for the author, including concerns about dual publication, research ethics, or publication ethics. (Please upload your review as an attachment if it exceeds 20,000 characters)

Reviewer #1: (No Response)

---

7. PLOS authors have the option to publish the peer review history of their article (what does this mean?). If published, this will include your full peer review and any attached files.

If you choose "no", your identity will remain anonymous but your review may still be made public.

**Do you want your identity to be public for this peer review?** For information about this choice, including consent withdrawal, please see our Privacy Policy.

Reviewer #1: No

---

[NOTE: If reviewer comments were submitted as an attachment file, they will be attached to this email and accessible via the submission site. Please log into your account, locate the manuscript record, and check for the action link "View Attachments". If this link does not appear, there are no attachment files.]

While revising your submission, please upload your figure files to the Preflight Analysis and Conversion Engine (PACE) digital diagnostic tool, https://pacev2.apexcovantage.com/. PACE helps ensure that figures meet PLOS requirements. To use PACE, you must first register as a user. Registration is free. Then, login and navigate to the UPLOAD tab, where you will find detailed instructions on how to use the tool. If you encounter any issues or have any questions when using PACE, please email PLOS at figures@plos.org. Please note that Supporting Information files do not need this step.
