# Supplementary material for: Influencing factor of COVID-19 vaccination trust and hesitancy in Wonju city, South Korea
Source: PLoS One. 2022 Nov 14;17(11):e0277016. doi: 10.1371/journal.pone.0277016 (PMC9662712; doi:10.1371/journal.pone.0277016)
Supplement: Attachment — Submitted filename: Response to Reviewers.docx [file pone.0277016.s004.docx]

**Comments to the Author**

**Authors**: First, we are very appreciated for your helpful review. I discussed with all authors for your comments. And we revised/corrected the context as all manuscript.

We attached the two manuscripts, 1) clean manuscript, 2) tracked manuscript. In the ’ 2) track manuscript’ file, our responses/revisions are highlighted using tracked system on word.

Thank you for review in advance.

Prof. Eun Woo Nam.

1. If the authors have adequately addressed your comments raised in a previous round of review and you feel that this manuscript is now acceptable for publication, you may indicate that here to bypass the “Comments to the Author” section, enter your conflict of interest statement in the “Confidential to Editor” section, and submit your "Accept" recommendation.

Reviewer #1: (No Response)

2. Is the manuscript technically sound, and do the data support the conclusions?

The manuscript must describe a technically sound piece of scientific research with data that supports the conclusions. Experiments must have been conducted rigorously, with appropriate controls, replication, and sample sizes. The conclusions must be drawn appropriately based on the data presented.

Reviewer #1: Partly

**Authors**: We corrected the context as a whole manuscript. Please review the attachment.

3. Has the statistical analysis been performed appropriately and rigorously?

Reviewer #1: N/A

4. Have the authors made all data underlying the findings in their manuscript fully available?

The [PLOS Data policy](http://www.plosone.org/static/policies.action#sharing) requires authors to make all data underlying the findings described in their manuscript fully available without restriction, with rare exception (please refer to the Data Availability Statement in the manuscript PDF file). The data should be provided as part of the manuscript or its supporting information, or deposited to a public repository. For example, in addition to summary statistics, the data points behind means, medians and variance measures should be available. If there are restrictions on publicly sharing data—e.g. participant privacy or use of data from a third party—those must be specified.

Reviewer #1: No

**Authors**: Data cannot be shared without permission by Wonju city hall publicly, South Korea.

Data are available from the Wonju city hall Institutional Data Access / Ethics Committee (contact via Eun Woo Nam) for researchers who meet the criteria for access to confidential data.

5. Is the manuscript presented in an intelligible fashion and written in standard English?

PLOS ONE does not copyedit accepted manuscripts, so the language in submitted articles must be clear, correct, and unambiguous. Any typographical or grammatical errors should be corrected at revision, so please note any specific errors here.

Reviewer #1: No

**Authors**

1. We corrected the sentence, context as a whole manuscript. We also were followed the instructions available at <https://journals.plos.org/plosone/s/submission-guidelines>.

2. Note that I have added line numbers per journal guidelines: "Include page numbers and line numbers in the manuscript file. Use continuous line numbers (do not restart the numbering on each page)."

3. Note that I have added line numbers per journal guidelines: "Include page numbers and line numbers in the manuscript file. Use continuous line numbers (do not restart the numbering on each page)."

4. (Abstract) I have removed this sentence “The Korean government aims to completely ……” from the abstract as it gives little significance to the background of the study; you may reserve this for the main body. Until then, it's best to be direct in your abstract. The statement "Social capital (SC) has been documented to effectively reduce the spread of diseases, including COVID-19" is enough to introduce your study's aim.

5. We checked the all reference.

6. Review Comments to the Author

Please use the space provided to explain your answers to the questions above. You may also include additional comments for the author, including concerns about dual publication, research ethics, or publication ethics. (Please upload your review as an attachment if it exceeds 20,000 characters)

Reviewer #1: (No Response)

7. PLOS authors have the option to publish the peer review history of their article ([what does this mean?](https://journals.plos.org/plosone/s/editorial-and-peer-review-process#loc-peer-review-history)). If published, this will include your full peer review and any attached files.


If you choose “no”, your identity will remain anonymous but your review may still be made public.


**Do you want your identity to be public for this peer review?** For information about this choice, including consent withdrawal, please see our [Privacy Policy](https://www.plos.org/privacy-policy).

Reviewer #1: No

[NOTE: If reviewer comments were submitted as an attachment file, they will be attached to this email and accessible via the submission site. Please log into your account, locate the manuscript record, and check for the action link "View Attachments". If this link does not appear, there are no attachment files.]

While revising your submission, please upload your figure files to the Preflight Analysis and Conversion Engine (PACE) digital diagnostic tool, <https://pacev2.apexcovantage.com/>. PACE helps ensure that figures meet PLOS requirements. To use PACE, you must first register as a user. Registration is free. Then, login and navigate to the UPLOAD tab, where you will find detailed instructions on how to use the tool. If you encounter any issues or have any questions when using PACE, please email PLOS at [figures@plos.org](mailto:figures@plos.org). Please note that Supporting Information files do not need this step.
