# Supplementary material for: Influencing factor of COVID-19 vaccination trust and hesitancy in Wonju city, South Korea
Source: PLoS One. 2022 Nov 14;17(11):e0277016. doi: 10.1371/journal.pone.0277016 (PMC9662712; doi:10.1371/journal.pone.0277016)
Supplement: Attachment — Submitted filename: 03-2.__Reviewer_3__Response_to_Reviewers.docx [file pone.0277016.s006.docx]

Response to reviewers

[Reviewer #3]

Dear Reviewer:

We appreciate your careful review of our manuscript. All authors have read and discussed your comments and revised/corrected the manuscript accordingly.

Please find attached two manuscripts, one is a clean manuscript and the other is a tracked manuscript, which contains all our revisions made using the “Track Changes” feature in Microsoft Word.

We are grateful for your comments that have helped us improve the quality of our manuscript. Thank you in advance for reviewing our responses and revisions.

Sincerely,

Prof. Eun Woo Nam

|  | Comments | Responses |
| --- | --- | --- |
| Title | Since the sample is exclusively collected from a specific study site, I suggest the authors include the study site in the title. | Thank you for your comment.  We have changed the title of our study as follows:  “Influencing factors of COVID-19 vaccine trust and hesitancy in Wonju city, South Korea” |
| Abstract | (Line 31) 2.642 -> 2.660 | The value has been revised. |
|  | (Line 34) 1.424 -> 1.400 | The value has been revised. |
| Introduction | Please update these statistics to the current situation. | We have updated the statistics to reflect the current status of COVID-19 in South Korea. |
|  | Is the target achieved? Please elaborate on this issue. | Thank you for your helpful comment.  We have updated the vaccination rate in South Korea as of September 2022. |
| Method | (Study Design) Please elaborate why this city was selected as the study site. | Thank you for your comments.  To address your concern, we have added the following sentence to the revised manuscript:  “In 2021, the city implemented a five-year health plan, which includes measures for COVID-19 vaccination. Hence, this study aims to investigate the association of COVID-19 vaccine trust and hesitancy with SC to present useful data for devising COVID-19 policies. The study population comprised Korean adult (≥ 18 years) residents of Wonju.” |
|  | (Data collection) Please elaborate how the sample selection process (randomly) was carried out in this study. As I understand, we must have a list of all population members to select the sample randomly. | Thank you for your helpful comments.  We have included the following sentence to our revised manuscript:  “In Step 2, households were randomly selected from the list of households of a village within each sample stratified by age and sex, and a questionnaire survey was administered to these households.” |
|  | (Data collection) Please report briefly the results of this pilot study in this paragraph. | Thank you for your comment.  We have added the following information regarding the pilot survey:  “A pilot survey was conducted in four regions on May 5, and the main survey was conducted from May 7 to 17. The pilot survey was conducted on 100 participants in two urban and two rural regions, and reliability (Cronbach’s alpha) was found to be .86. Further, the content and construct validities of the questionnaire were determined based on the results of the pilot survey.” |
|  | (Data collection) Add the percentage of the total sample examined in the analysis i.e. 1,096/1,248x100=87.82%. | Thank you for your comment.  As instructed, we have added this information in our revised manuscript. |
|  | (Statistical Analysis) Besides the box plot, the authors also performed comparison analysis to check the differentiation of the SC scores among categories of a variable in Figure 1. Please report the test in this section. | Thank you for your comment.  We have added the following sentence:  “Further, the differences in SC according to participants’ characteristics were analyzed using t-tests.” |
|  | (Statistical Analysis) I think the authors need to elaborate the univariate and multivariate analysis in this section. Moreover, most studies employed a univariate analysis as a way to select a variable that will be included in the multivariate model. Only significant variables in univariate analysis would be included in the multivariate analysis.  See these studies as examples.  https://www.frontiersin.org/articles/10.3389/fpubh.2020.00381/full  https://narraj.org/main/article/view/55/44 | Thank you for your comments, which we agree with.  As you have correctly pointed out, this statistical method suffers from the following issue:   - Only significant variables in univariate analysis would be included in the multivariate analysis.   The following Lance Public Health article conducts statistical analysis similar to ours:   - <https://doi.org/10.1016/S2468-2667(21)00012-8>   As we are sure you are aware, the study of COVID-19 vaccine needs more identifiable evidence (factors).  Therefore, while we agree with you, we request that you consider the statistical analysis conducted in this study as is. |
| Results | (Participant characteristics) Based on Table 1, it seems that the most common age group was >= 60 years old (22.0%). | Apologies for the error and thank you for bringing this to our notice.  We have corrected the typo. |
|  | (Participant characteristics) Why were the characteristics based on household income not highlighted in this paragraph? | Thank you for your question.  We have added the following sentence to the revised manuscript:  “The most common household income was ≥ 5 million KRW (22.4%) (Table 1).” |
|  | (Participant characteristics - Figure 1) Figure 1 is unclear (low resolution) and hard to read. Moreover, please include the p-values of all variables in the top or below the box plot in Figure 1. | Thank you for your comments.  We have uploaded an alternative high-quality file.  You can access this file by clicking on the link provided in the top right corner of the figure below.  (URL: <https://www.editorialmanager.com/pone/download.aspx?id=31783918&guid=c12faffc-27ea-491c-af18-d20045fc4237&scheme=1>)   |
|  | (Participant characteristics) p-value will never equal zero. It should be written as "<0.001". | Thank you for your comments.  We have replaced “<.0000” with “<0.001” in the revised manuscript. |
|  | (Participant characteristics) Based on Figure 1, the respondent who did not trust Covid-19 vaccine seems to have lower SC scores. Moreover, how about the characteristics based on vaccine hesitancy, Covid-19 screening, and area? Only those variables were not elaborating in this paragraph. | Thank you for your helpful comment.  We conducted the t-test again according to your suggestion.  Consequently, we have added the following sentence to our revised manuscript:  “Individuals with as opposed to without vaccine hesitancy showed significantly higher SC (*p*<.001). Urban dwellers displayed higher SC than rural dwellers but not to a significant extent (*p*=.945) (Fig 1).” |
|  | P<0.000 -> p<.0001 | Thank you for pointing this error out. We have replaced “<.0000” with “<0.001” in the revised manuscript. |
| Discussion | Please include these studies.  https://www.frontiersin.org/articles/10.3389/fpubh.2020.00381/full  https://narraj.org/main/article/view/85 | Thank you for the recommendations.  We have referred to and included the following study in our paper:  <https://www.frontiersin.org/articles/10.3389/fpubh.2020.00381/full>  Please check reference 23.  We have also added the following sentences to our revised manuscript:  “On the other hand, a Southeast Asian study reported that older adults are indeed vulnerable to COVID-19, but most of this population is retired and spends most of its time at home. Therefore, the study argued that the older adult population is at a lower risk of exposure to COVID-19 and, consequently, shows lower vaccine acceptance [23].” |
