# Supplementary material for: Influencing factor of COVID-19 vaccination trust and hesitancy in Wonju city, South Korea
Source: PLoS One. 2022 Nov 14;17(11):e0277016. doi: 10.1371/journal.pone.0277016 (PMC9662712; doi:10.1371/journal.pone.0277016)
Supplement: Attachment — Submitted filename: 3-2. [#Reviewer Comments] response to reviewer.docx [file pone.0277016.s003.docx]

[#Reviewer 1] 07 Juine 2022

We would like to appreciate for your helpful comments and suggestion for our manuscript. We consider all comments and revised our manuscript according to 1^st^ comments by reviewers. we attached 2 manuscripts; 1) Clean manuscripts, 2) Manuscripts where we changed using ‘Track Change’ tool in word (marked in red).

| **No** | **Reviewer’s comments** | **Response** |
| --- | --- | --- |
|  | I read the manuscript entited "Association between social capital and COVID-19 vaccine trust and hesitancy". The concept is interesting but already it is old idea. I see many article in online about the COVID-19 vaccine hesitency. However, most of them are missing in present manuscript. Therefore, authors need to improve introduction and discussion based on present published articles. See some specific comments bellow: | First, we very much appreciate your helpful comments.  We agree this concept is an old idea.  But, please consider that we submitted in Sep 2021.  This manuscript improved quality due to your good comment.  Thanks. |
|  | consent from individual was missing. If you have that, add in the data colection section | We very agree with your comment.  Consent process is very important.  Therefore, we added the this sentence ;  We obtained informed consent in written form from all the respondents. It must be noted that special notifications on the respondents’ rights to refuse to answer questions were sent to the participants. |
|  | Table 1: Age categories should be minimized, so many catagories is meaningless. So I suggested to have only 3/4 categories of age. | Thank you for your comment.  All coauthors discussed for age categories as following your comments.  As results, we categorized like ;  : 20-29/30-39/40-49/50-59/over 60.  Thanks. |
|  | How you divided the household incomes? You need to add references in the method section, how you divided the income division? Accordingly, re-arrange the Table 1 based on income categories! | We added the this sentence ;  House income was classified as less than 1 million, 1-1.99 million, 2-2.99 million or more, 3-3.99 million or more, 4-4.99 million or more, and 5 million or more. This was based on the classification table of the Korea National Statistical Office. . |
|  | Rearrange Table 2 and 3 based on re-arranged categories of age and household incomes. | Thank you for comment.  We rearrange the Table 2, Table 3.  In our opinion, this manuscript results improved by re-arrange the category.  Thank you. |
|  | What is your key findings from your study? Need to explain key messages in the conclusion part. Based on the key message, what is your recommendations. | It is very important comment and helpful comment for improving the article quality.  Key finding is “This study confirmed that SC is a predictor of COVID-19 vaccine trust and hesitancy, and that people with higher SC have greater trust in COVID-19 vaccines and are more willing to be vaccinated.”  And We recommended by this sentence on end of conclusion ;  To this end, the Korean government will need to transparently provide reliable COVID-19 information through various channels such as the media and the Internet through COVID-19 experts and celebrities to increase its credibility. |
|  | In Figure 1, you need to adjust age, and household incomes. | Thank you for your very good comment.  We re-made and re-designed the figure 1. |
| We are very appreciated your professional comment.  Thank you again. Again, | | |

**Note: the modification we made are marked using ‘Track Change’ tool in word in the revised manuscript**
